# Investigation of the impact of the NICE guidelines regarding antibiotic prophylaxis during invasive dental procedures on the incidence of infective endocarditis in England: an electronic health records study

**DOI:** 10.1186/s12916-020-01531-y

**Published:** 2020-04-02

**Authors:** T. Phuong Quan, Berit Muller-Pebody, Nicola Fawcett, Bernadette C. Young, Mehdi Minaji, Jonathan Sandoe, Susan Hopkins, Derrick Crook, Timothy Peto, Alan P. Johnson, A. Sarah Walker

**Affiliations:** 1 National Institute for Health Research (NIHR) Health Protection Research Unit on Healthcare Associated Infections and Antimicrobial Resistance, John Radcliffe Hospital, Microbiology Level 7, Headley Way, Oxford, OX3 9DU UK; 2 Nuffield Department of Medicine, University of Oxford, John Radcliffe Hospital, Headley Way, Oxford, OX3 9DU UK; 3 NIHR Biomedical Research Centre, Oxford, OX3 9DU UK; 4 National Infection Service, Public Health England, Colindale, London, UK; 5 Oxford University Hospitals NHS Foundation Trust, John Radcliffe Hospital, Headley Way, Oxford, OX3 9DU UK; 6 Department of Microbiology, Leeds Teaching Hospitals NHS Trust and University of Leeds, Leeds, LS1 3EX UK

**Keywords:** Infective endocarditis, Dental procedures, Antibiotic prophylaxis, Electronic health records, EHR

## Abstract

**Background:**

Infective endocarditis is an uncommon but serious infection, where evidence for giving antibiotic prophylaxis before invasive dental procedures is inconclusive. In England, antibiotic prophylaxis was offered routinely to patients at risk of infective endocarditis until March 2008, when new guidelines aimed at reducing unnecessary antibiotic use were issued. We investigated whether changes in infective endocarditis incidence could be detected using electronic health records, assessing the impact of inclusion criteria/statistical model choice on inferences about the timing/type of any change.

**Methods:**

Using national data from Hospital Episode Statistics covering 1998–2017, we modelled trends in infective endocarditis incidence using three different sets of inclusion criteria plus a range of regression models, identifying the most likely date for a change in trends if evidence for one existed. We also modelled trends in the proportions of different organism groups identified during infection episodes, using secondary diagnosis codes and data from national laboratory records. Lastly, we applied non-parametric local smoothing to visually inspect any changes in trend around the guideline change date.

**Results:**

Infective endocarditis incidence increased markedly over the study (22.2–41.3 per million population in 1998 to 42.0–67.7 in 2017 depending on inclusion criteria). The most likely dates for a change in incidence trends ranged from September 2001 (uncertainty interval August 2000–May 2003) to May 2015 (March 1999–January 2016), depending on inclusion criteria and statistical model used. For the proportion of infective endocarditis cases associated with streptococci, the most likely change points ranged from October 2008 (March 2006–April 2010) to August 2015 (September 2013–November 2015), with those associated with oral streptococci decreasing in proportion after the change point. Smoothed trends showed no notable changes in trend around the guideline date.

**Conclusions:**

Infective endocarditis incidence has increased rapidly in England, though we did not detect any change in trends directly following the updated guidelines for antibiotic prophylaxis, either overall or in cases associated with oral streptococci. Estimates of when changes occurred were sensitive to inclusion criteria and statistical model choice, demonstrating the need for caution in interpreting single models when using large datasets. More research is needed to explore the factors behind this increase.

## Background

Infective endocarditis is an uncommon but serious infection, for which the evidence for giving antibiotic prophylaxis to people undergoing invasive dental procedures is inconclusive. In March 2008, the National Institute for Health and Care Excellence (NICE) issued guidelines recommending that antibiotic prophylaxis during invasive dental procedures should no longer be routinely offered to people at risk of infective endocarditis in England [1]. This was in contrast to American Heart Association (AHA) [2] and European Society of Cardiology (ESC) [3] guidelines issued around the same time, which continued to recommend antibiotic prophylaxis in certain high-risk cases, e.g., patients with prosthetic heart valves or who had had infective endocarditis previously. Although much research on the impact of guideline changes on the incidence of infective endocarditis has been conducted internationally [4–15], and in particular a study in England which showed an increase in cases following the NICE guideline change [6], no consensus has been reached, and in a 2016 update to their guidelines [16] NICE reaffirmed their previous position, while clarifying that doctors and dentists should still apply their clinical judgement on a case by case basis.

A recent study of ICD-10 (International Classification of Diseases, Tenth Revision) diagnosis codes used to represent infective endocarditis cases at two large English hospital trusts [17] concluded that the inclusion criteria for observational studies using electronic health records (EHRs) need to be selected very carefully, as, even when specific diagnostic codes are chosen with care, individual records may still not always represent confirmed clinical cases. To build on this, we conducted a range of analyses using national EHR data on infective endocarditis in England, in particular investigating whether changes in incidence could be detected around the change in NICE guidelines or at other times, and assessing the impact of inclusion criteria and statistical model choice on inferences drawn about timing and types of change. We also linked EHR data to national microbiology data to analyse trends in the microorganisms isolated from blood during each infective endocarditis episode, in particular those genera or species known to commonly colonise the oropharynx, which to our knowledge has not previously been done in England.

## Methods

### Incidence of infective endocarditis

To measure national incidence of infective endocarditis between April 1998 and March 2017 inclusive, we used data from the Admitted Patient Care dataset from Hospital Episode Statistics (HES), which contains details of all inpatient admissions to NHS hospitals in England, with clinical diagnoses recorded using ICD-10 codes. In HES, diagnosis codes are recorded against finished consultant episodes, so after identifying all episodes that contained a code for infective endocarditis, we concatenated adjoining episodes (including where patients transferred between different providers) into continuous inpatient spells [18] (also known as “superspells”). To identify incident cases of infective endocarditis, we used three different inclusion criteria (designated A–C), reflecting possible differences in sensitivity:

- Criteria A: At least one of the ICD-10 codes: I33.0, I33.9, I39.0, I39.8, I01.1, B37.6, or T82.6 in any diagnosis field, or I38 in the primary diagnosis field, in any episode in a superspell, where the patient was not discharged alive within 2 days, and excluding any readmissions within 30 days (using the HES patient ID as the patient identifier)
- Criteria B: ICD-10 code I33.0 in the primary diagnosis field, in any episode in a superspell, where the patient was not discharged alive within 2 days, excluding any readmissions within 30 days (using the HES patient ID as the patient identifier), and excluding elective admissions
- Criteria C: ICD-10 code I33.0 in the primary diagnosis field, in any episode in a superspell, excluding those with an admission method of “Elective - waiting list”

Criteria A and B were shown by Fawcett et al. [17] to represent the true number of infective endocarditis cases more accurately than simpler criteria, with Criteria A maximising sensitivity plus positive predictive value (PPV) and Criteria B maximising specificity plus PPV, while Criteria C was that employed by Dayer et al. [6], the most prominent prior study based on national HES data.

Using annual population estimates from the Office for National Statistics [19], we applied two different methods to control for changes in the underlying population: (1) by dividing the monthly cases by the total population of England (using linear interpolation between each annually estimated figure to avoid sudden jumps in the denominator) and (2) by direct standardisation to the (5-year) age and sex distribution of England in 1998.

We also calculated incidence of infective endocarditis cases in high-risk individuals (out of the same underlying denominator populations), defining “high risk” by the AHA [2] and ESC [3] guidelines, i.e. cases where there had been a previous admission for infective endocarditis (using the same case definitions within each criteria) or pre-existing prosthetic valve or congenital heart disease (using the same coding criteria implemented by Dayer et al. (Additional file 1: Table S1)), and separately in cases with current or previous codes reflecting illicit drug use (F11 (opioids), F12 (cannabinoids), F14 (cocaine), F19 (multiple or other psychoactive substances), T40 (poisoning by narcotics/psychodysleptics)). (While it is specifically intravenous drug use that results in an increased risk for endocarditis, there are currently no diagnosis codes that directly represent this, and therefore illicit drug use was used as a proxy.) Since these methods are dependent on data from previous admissions, we only calculated incidence from year end 1999 onwards, to allow for a “burn-in” time of one calendar year.

### Causal organisms

We identified potential causal organisms using secondary diagnosis codes that were present in the same consultant episode(s) as the code(s) for infective endocarditis within a superspell, and categorised these into three overall groups: *Streptococcus* species, *Staphylococcus* species, and other/unnamed species (Additional file 1: Table S2). If more than one organism code was present in a superspell (e.g. if a superspell consisted of multiple episodes with different secondary organism codes and/or an episode included more than one organism code), we included them all.

Since ICD-10 codes do not distinguish between infection with oral and non-oral streptococci, we further matched the HES records to microbiological test results in Public Health England’s Second Generation Surveillance System (SGSS), which receives microbiology results from > 98% of hospital laboratories in England. Organisms from blood specimens recorded in SGSS were matched to episodes in HES that contained an infective endocarditis diagnosis code based on NHS number and specimen date between 7 days before episode start date up to episode end date, by the data manager at Public Health England who had authorisation to view personal identifiable data (under Regulation 3 of the Health Service (Control of Patient Information) Regulations 2002). If more than one SGSS record was matched within a superspell, we included them all. We considered the organisms from SGSS and HES to be in agreement for an infective endocarditis case if at least one organism from each source was present and belonged to the same overall group (as defined above). We modelled overall trends in organism group proportions and in SGSS/HES agreement using Poisson regression (or negative binomial regression when there was evidence of overdispersion) with the following denominators as exposure variables: for organisms based on HES diagnosis codes, we controlled for the denominator of cases with *any* organism coded in HES; for SGSS/HES agreement, we controlled for the denominator of cases that had any organism present both in SGSS and in HES; and for organisms based on SGSS records, we controlled for the denominator of cases with any match to an organism record in SGSS. Models using SGSS-linked data were restricted to dates after October 2002, when there were consistently at least 10 infective endocarditis cases matched to an SGSS organism per month. We further categorised SGSS organisms into nine subgroups: oral streptococci, pyogenic streptococci, Group D streptococci, other streptococci, HACEK (a group of fastidious Gram-negative bacteria that are a known cause of infective endocarditis) [20], enterococci, *Staphylococcus aureus*, coagulase-negative staphylococci, and “Other” (Additional file 1: Table S3).

### Temporal association between infective endocarditis incidence and change in prophylaxis guideline

Date-based interventions are often assessed using an interrupted time-series analysis, comparing the trend in incidence before and after the intervention date. However, when the overall trend is non-linear, this methodology is biased towards finding a positive result (see Additional file 1: Table S4). To avoid this, we instead fitted a range of models to identify those that fitted the data the best, to investigate the evidence supporting a change in incidence of infective endocarditis following the guideline change as opposed to at other time points. We systematically fitted piecewise linear Poisson (or negative binomial) regression models to the raw monthly cases (with and without adjusting for total population as an exposure variable), as well as to the standardised monthly cases. We fitted four different types of model: (1) a single overall trend, (2) two (potentially) different trends before and after a single change point, (3) two (potentially) different trends before and after a single change point plus a step change at that point, (4) a single step change only, with no trend either before or after the change. We used a grid search algorithm, considering single change points at each month from October 1997 to September 2016 inclusive, and selecting the best-fitting model and “month of change” by Akaike Information Criterion (AIC) [21]. Uncertainty intervals were estimated as the range of dates within a difference in AIC of < 3.84 from the model with the best-fitting date, taking the minimum and maximum dates even if there were non-contiguous ranges of dates within this threshold. The same range of models were fitted to the monthly proportion of infective endocarditis cases that contained a HES streptococcal code (out of the total number of infective endocarditis cases that contained any HES organism code), as well as to the monthly proportion of infective endocarditis cases that matched to an oral *Streptococcus* in SGSS (out of the total number of infective endocarditis cases that matched to any organism record in SGSS, and restricting this latter search to change points between April 2003 and September 2016 (since data prior to October 2002 were excluded from the models due to low numbers of cases matching to SGSS (see above)). These proportions were modelled as monthly cases associated with the particular type of organism against time as the independent variable, with the relevant denominator included as an exposure variable.

Additionally, instead of making an a priori assumption of fixed incidence rates before and after a single change point, we applied a non-parametric LOWESS smoother [22] to visually inspect trends. We compared these to what would be expected under a hypothesis that dental prophylactic antibiotic prescribing was protective against the development of infective endocarditis (both for total cases and for the proportion of cases linked to oral streptococci), against an assumed background of linearly increasing cases unrelated to dental prophylactic antibiotic prescribing. To confirm that the change in guideline resulted in reduced dental prophylactic antibiotic prescribing, we downloaded annual data on two types of prescriptions known to be used almost exclusively [6] for dental prophylaxis in the community (3 g doses of amoxicillin and 600 mg doses of clindamycin) from NHS Digital [23]. We plotted annual numbers of 3 g amoxicillin doses only, as it was not possible to distinguish 600 mg doses of clindamycin (as opposed to other dose strengths) from the data, although it has previously been shown that the latter form only around 25% or less of prophylactic prescribing and follow the same pattern as 3 g amoxicillin doses [6].

All analyses were conducted using Stata v15.1 (StataCorp).

## Results

### Incidence of infective endocarditis

**Fig. 1:**
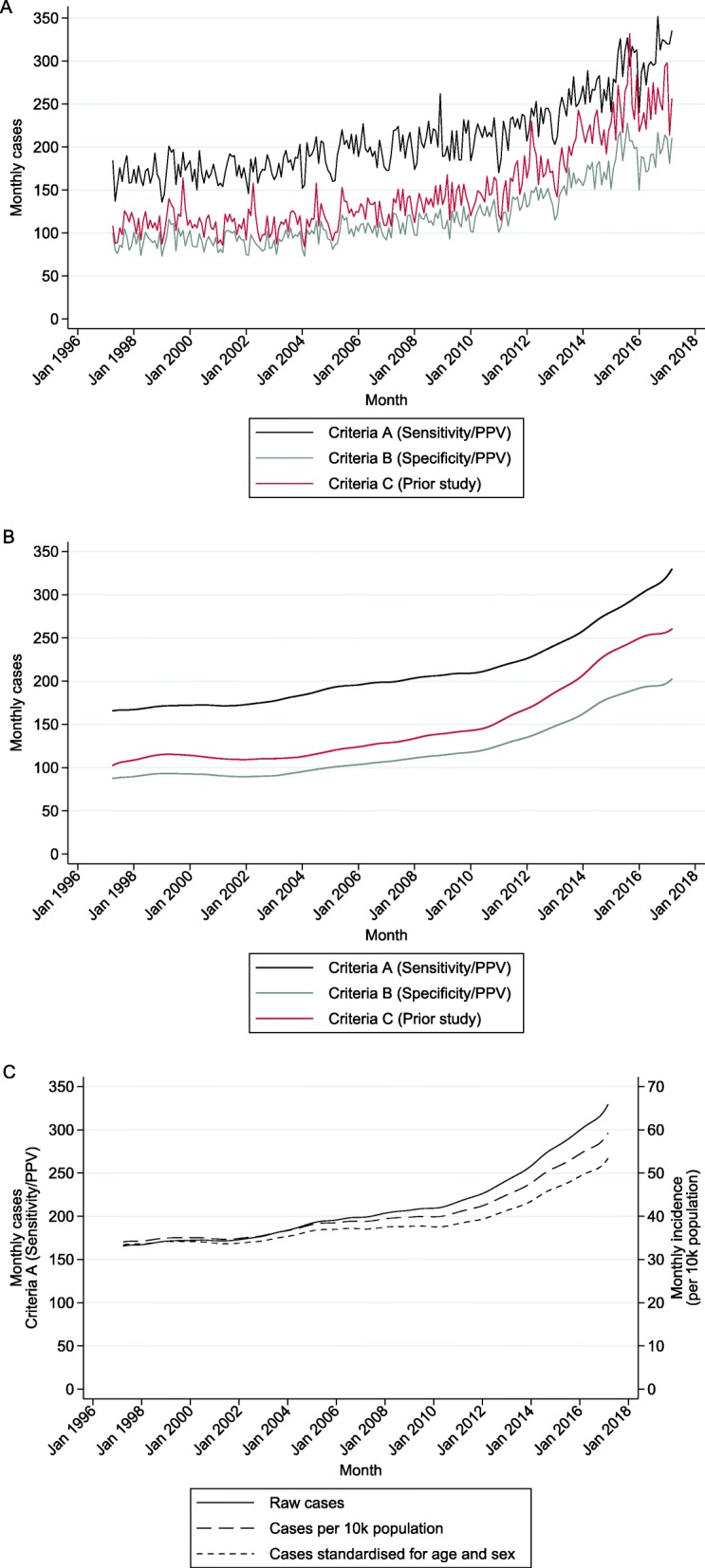
Monthly cases of infective endocarditis. Different coloured lines represent different inclusion criteria. **a** Raw cases. **b** Raw cases after applying a non-parametric LOWESS smoother. **c** Effect of applying different methods to adjust for changes in population, shown here for all cases using Criteria A, but results are similar for all three criteria (see Additional file 2: Figure S1)

The incidence of infective endocarditis in England increased between April 1998 and March 2017, irrespective of which of the three criteria we used to measure it (Fig. 1a). Annual numbers of cases and incidence rates can be seen in Table 1. Criteria A (optimised for sensitivity/PPV) produced the highest numbers of cases overall, while Criteria B (optimised for specificity/PPV) produced the lowest numbers of cases. The trends using Criteria A and B appeared very similar throughout the entire period, whereas the trend based on Criteria C (used by the largest prior English study) appeared to increase more rapidly compared to the other two Criteria from around 2010 onwards (Fig. 1b). Controlling for changes in population attenuated the yearly increases but did not change the overall trend pattern (Fig. 1c, Additional file 2: Figure S1).

**Table 1:** Annual numbers of cases of infective endocarditis based on the three different criteria, along with incidence rates and age and sex distribution

Year end	Criteria A (optimising sensitivity/PPV)				Criteria B (optimising specificity/PPV)				Criteria C (used in prior study [6])			
	No. of cases	Incidence rate per 1 million population	Percentage male	Median age (IQR)	No. of cases	Incidence rate per 1 million population	Percentage male	Median age (IQR)	No. of cases	Incidence rate per 1 million population	Percentage male	Median age (IQR)
1998	2010	41.3	60.8	65 (49–75)	1079	22.2	65.2	63 (46–74)	1304	26.8	65.1	63 (46–73)
1999	1968	40.3	61.5	65 (50–75)	1066	21.8	63.7	63 (45–73)	1297	26.6	65.6	62 (44–72)
2000	2157	44.0	61.8	66 (50–76)	1173	23.9	65.2	64 (47–75)	1479	30.2	64.5	61 (42–74)
2001	1995	40.5	63.6	64 (49–75)	1043	21.2	68.0	63 (47–74)	1256	25.5	68.4	62 (43–74)
2002	2102	42.6	59.8	66 (48–76)	1126	22.8	61.9	65 (47–76)	1357	27.5	61.8	64 (45–75)
2003	2086	42.0	62.4	66 (48–77)	1035	20.8	67.1	66 (47–75)	1295	26.1	67.2	64 (43–74)
2004	2180	43.7	65.7	65 (47–75)	1137	22.8	69.1	64 (43–75)	1338	26.8	70.7	63 (42–74)
2005	2228	44.4	63.2	65 (46–76)	1174	23.4	66.5	64 (44–75)	1384	27.6	67.5	63 (43–74)
2006	2463	48.7	65.1	65 (44–77)	1274	25.2	68.1	63 (42–75)	1530	30.2	69.5	62 (41–75)
2007	2268	44.5	65.6	66 (47–77)	1224	24.0	69.3	64 (45–76)	1472	28.9	69.3	63 (45–76)
2008	2456	47.8	66.4	66 (48–77)	1337	26.0	70.8	64 (46–76)	1620	31.5	71.0	63 (45–75)
2009	2531	48.9	65.9	65 (47–76)	1385	26.7	68.2	63 (43–75)	1690	32.6	69.5	62 (43–75)
2010	2452	47.0	64.4	67 (50–78)	1364	26.1	68.2	66 (49–77)	1655	31.7	69.4	66 (46–76)
2011	2546	48.4	66.1	66 (50–77)	1470	27.9	68.4	66 (49–77)	1739	33.1	67.6	65 (48–76)
2012	2703	50.9	66.1	66 (50–77)	1595	30.0	69.4	66 (51–77)	2035	38.3	69.1	64 (46–75)
2013	2773	51.8	67.0	67 (50–78)	1682	31.4	69.2	66 (49–77)	2074	38.8	69.9	65 (46–77)
2014	3105	57.6	65.8	67 (49–78)	1924	35.7	67.3	66 (47–77)	2466	45.8	71.4	64 (45–76)
2015	3184	58.6	65.8	67 (50–78)	2054	37.8	67.7	66 (49–78)	2645	48.7	68.8	63 (47–76)
2016	3617	66.0	65.9	67 (48–78)	2384	43.5	67.4	66 (47–77)	3055	55.7	68.9	65 (47–77)
2017	3746	67.7	67.2	67 (48–78)	2325	42.0	69.8	67 (48–78)	3061	55.4	70.8	66 (46–76)

**Fig. 2:**
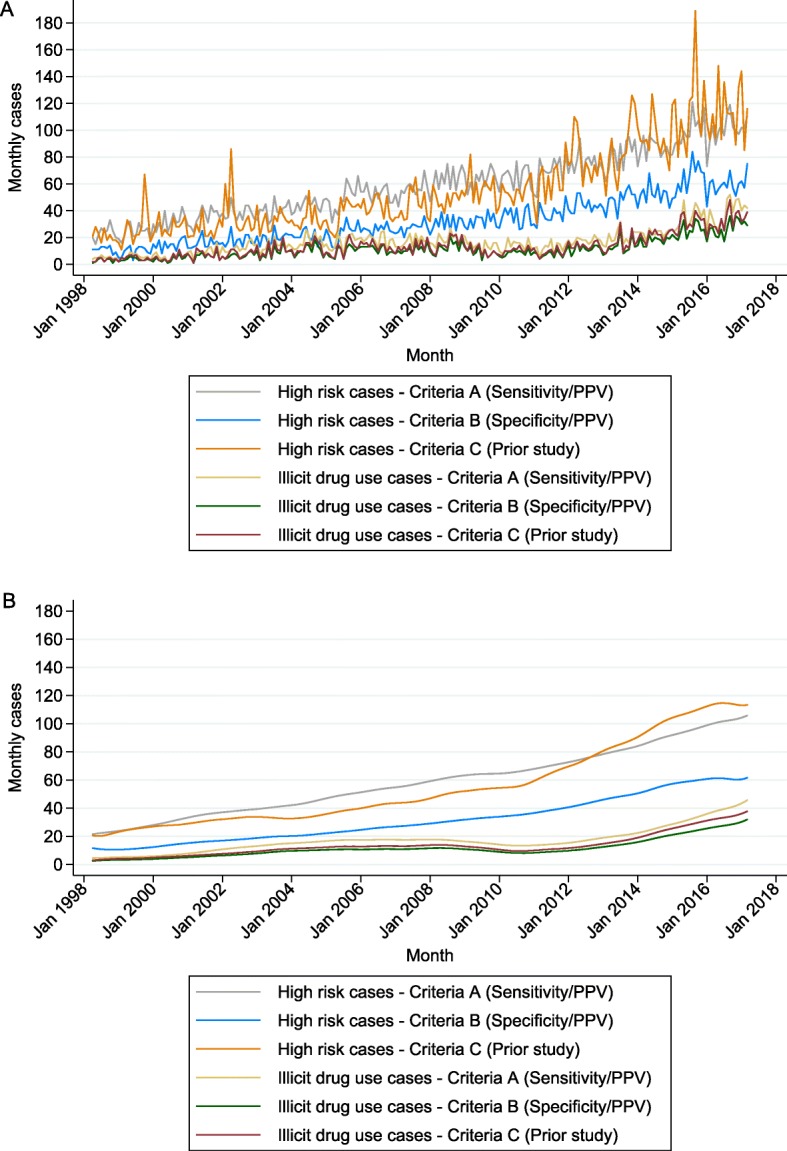
Monthly cases of infective endocarditis in individuals identified as high-risk or as illicit drug users. Different coloured lines represent different inclusion criteria. **a** Raw cases. **b** Raw cases after applying a non-parametric LOWESS smoother

High-risk individuals comprised 13,581/50570 (27%), 7286/28851 (25%), and 12,873/35752 (36%) cases for criteria A, B, and C respectively. Incidence of infective endocarditis in “high-risk” individuals also increased steadily (Fig. 2a), with the same divergence of Criteria C from the other two Criteria in around 2010 (Fig. 2b). Individuals with a history of illicit drug use comprised 3927/50570 (8%), 2590/28851 (9%), 3106/35752 (9%) cases for criteria A, B, and C respectively. Numbers of infective endocarditis cases in these individuals followed a slightly different pattern, increasing up until around 2008, dipping slightly until 2011, then increasing again more rapidly to levels in 2017 that were more than double the number at the earlier peak in 2008 (Fig. 2b). Trends in cases when excluding these individuals were similar to trends in overall cases (Additional file 2: Figure S2).

### Causal organisms

**Fig. 3:**
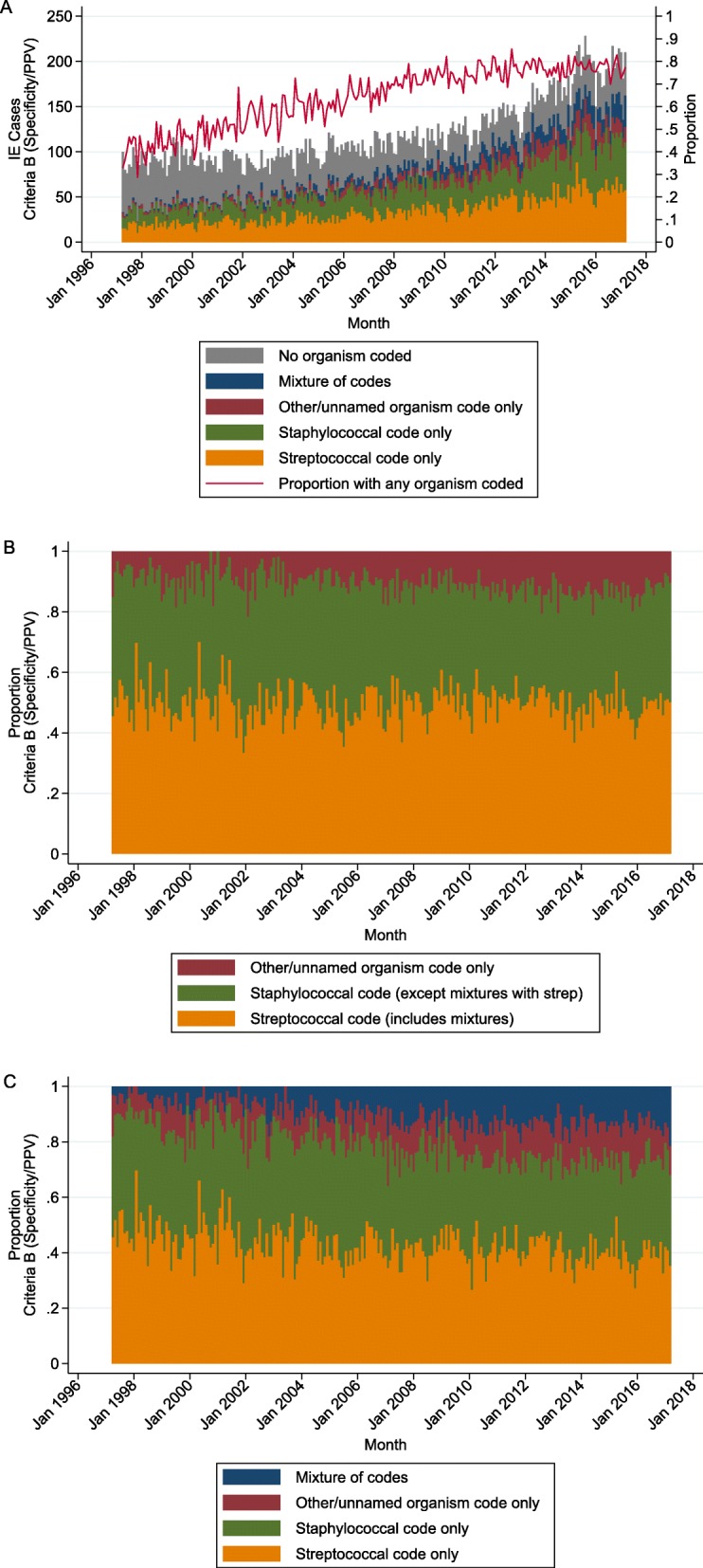
Causative organism based on secondary diagnosis codes in HES, using Criteria B (optimised for specificity/PPV). (For Criteria A and C, see Additional file 2: Figures S3–5). **a** Monthly infective endocarditis cases according to corresponding organism codes (in same consultant episode), along with overall proportion of cases with any organism coded. **b** Of infective endocarditis cases with an organism code present, proportion that were coded as streptococcal, staphylococcal, or other/unnamed, including mixtures. **c** Of infective endocarditis cases with an organism code present, proportion that were coded exclusively as streptococcal, staphylococcal or other/unnamed, or else with a mixture of codes

Considering Criteria B (i.e. optimised for specificity/PPV) first, since this minimises inclusion of false-positive cases, 19,290/28851 (67%) infective endocarditis cases contained a secondary diagnosis code for an organism in HES. The proportion of infective endocarditis cases with a secondary diagnosis code for an organism increased from around 40% in 1997 to roughly 75% in 2011, plateauing thereafter (Fig. 3a). Out of those with an organism coded, 9533 (49%) contained a code for streptococcal species (including mixtures) and 8244 (43%) contained a code for staphylococcal species (including mixtures), with no evidence of overall trend across the time period for these proportions (annual incidence rate ratio (aIRR) = 1.00 (95% CI 1.00, 1.00), *p* = 0.56; aIRR = 1.00 (0.99, 1.00), *p* = 0.06 respectively) (Fig. 3b). A total of 3908 (20%) cases contained a code for a different or unnamed organism (including mixtures), and this proportion increased over the period (aIRR = 1.05 (1.04, 1.05), *p* < 0.001). A total of 2250 (12%) cases had a mixture of organism codes, and this proportion increased over the period (aIRR = 1.05 (1.04, 1.06), *p* < 0.001) (Fig. 3c), while the proportion of cases coded exclusively as streptococcal or staphylococcal species decreased slightly over time (aIRR = 0.99 (0.99, 0.99), *p* < 0.001; aIRR = 0.99 (0.99, 0.99), *p* < 0.001 respectively). Patterns were similar for Criteria A and C (Additional file 2: Figures S3-S5).

**Fig. 4:**
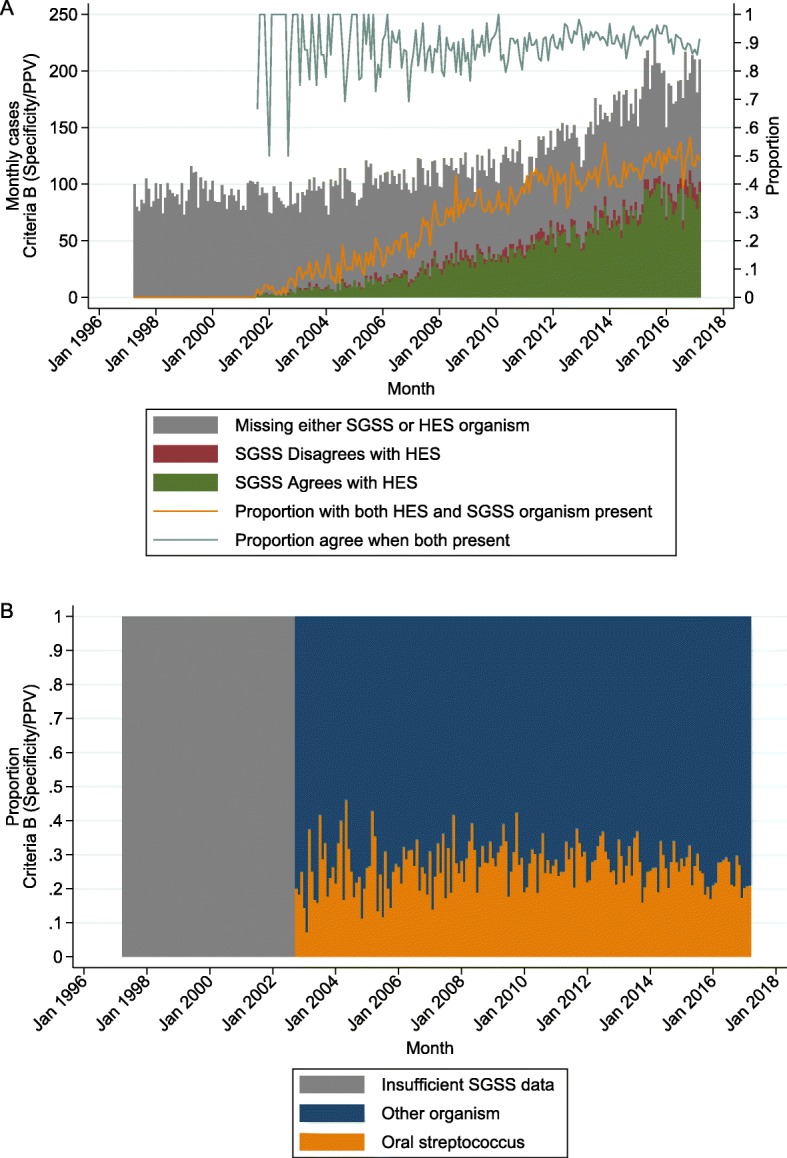
Causative organism based on SGSS, using Criteria B (optimised for specificity/PPV). (For Criteria A and C, see Additional file 2: Figures S6-S7). **a** Monthly agreement of SGSS organism compared to HES organism code, based on 3 groups: streptococcal, staphylococcal, other/unnamed. **b** Of all infective endocarditis cases that were matched to an organism in SGSS, proportion that were classed as oral streptococci

The proportion of Criteria B cases with both an organism code in HES and a microbiological record in SGSS increased from zero in 2001 to around 50% in 2017 (Fig. 4a). In cases where an organism was recorded in both HES and SGSS, 7095/7882 (90%) agreed at the overall group level (streptococcal, staphylococcal or other/unnamed species), with a modestly increasing trend over time (aIRR = 1.04 (1.02, 1.05), *p* < 0.001). Of the 10% (*n* = 787) of cases that disagreed, 463 (59%) contained a streptococcal code in HES and matched to an enterococcal record in SGSS. Of the 2229/19290 (12%) cases where HES only indicated other/unnamed species, 1188 (53%) did not match to any records in SGSS, 114 (5%) matched to a streptococcal record, 105 (5%) to a staphylococcal record, and 895 (40%) to other species (including 506 (23%) enterococci). Again, patterns were similar for Criteria A and C (Additional file 2: Figure S6).

Of all Criteria B cases that were matched to an organism in SGSS, 2855/10715 (27%) were identified as oral streptococci, and there was no evidence that this proportion changed over time (aIRR = 0.99 (0.98, 1.00), *p* = 0.08) (Fig. 4b). This pattern was similar for Criteria A and C (Additional file 2: Figure S7), and there was no qualitative difference in behaviour between different organism subgroups (Additional file 2: Figure S8).

### Temporal association between infective endocarditis incidence and change in prophylaxis guideline

As expected given the non-linear changes in incidence across the study period (Fig. 1), regression models testing for a difference in trend before and after a fixed date showed a bias towards a positive result; e.g. for Criteria A, a statistically significant (*p* < 0.05) increase in trend was found after 230 of the 238 possible dates tested across the period (Additional file 1: Table S4). The eight non-significant dates were all at the extreme ends of the study period (and were a consequence of wide confidence intervals due to the small number of data points at the extreme ends as opposed to the before vs after trend estimates being closer).

When considering the best-fitting models of each type (see “Methods”), December 2010, July 2011, and June 2011 were identified as the most likely month of change in incidence trends for Criteria A, B, and C respectively (34–39 months after the guideline change) (Table 2). For high-risk cases, the most likely month of change was variously identified as January 2000, September/October 2001, June 2002, or May 2015. Models which allowed for a different trend both before and after a change point fitted better than the models which enforced a zero trend or allowed no change point.

**Table 2:** Variation in goodness of fit and optimal change point based on different models for incidence of infective endocarditis, according to different criteria. For each model type, the month of change which gives the best model fit is shown, with best overall models shown in bold italics. AIC measures model goodness of fit (the lower the value the better the fit, within each set of inclusion criteria and method of population adjustment)

			Raw cases		Cases per 10 k population		Cases standardised by age and sex	
Inclusion criteria		Model type	AIC	Month of change (uncertainty interval)	AIC	Month of change (uncertainty interval)	AIC	Month of change (uncertainty interval)
All cases	Criteria A (Sensitivity/PPV)	***One change in trend, with step***	***2050.6***	***Dec 2010 (Jan 2009–Apr 2013)***	***2049.3***	***Dec 2010 (Jan 2009–Jul 2013)***	***2010.2***	***Dec 2010 (Jan 2009–Jul 2013)***
		One change in trend, no step	2050.6	Apr 2011 (Jul 2010***–***Jun 2012)	2049.9	May 2011 (Aug 2010***–***Aug 2012)	2011.6	Jan 2012 (Oct 2010***–***Mar 2013)
		Single overall trend	2140.6	NA	2132.2	NA	2080.1	NA
		No trend, with step	2245.6	Jun 2011 (Jun 2011***–***Sep 2011)	2176.3	Mar 2013 (Mar 2013***–***Apr 2013)	2096.6	Mar 2013 (Mar 2013***–***Apr 2013)
	Criteria B (Specificity/PPV)	***One change in trend, with step***	***1875.3***	***Jul 2011 (Nov 2008–Aug 2011)***	***1872.4***	***Jul 2011 (Jan 2009–Aug 2011)***	***1830.4***	***Jul 2011 (Jan 2009–Sep 2011)***
		One change in trend, no step	1875.5	Sep 2009 (Jun 2008***–***Aug 2010)	1873.3	Nov 2009 (Sep 2008***–***Oct 2010)	1831.1	Jan 2010 (Oct 2008***–***Jan 2011)
		Single overall trend	1996.5	NA	1987.3	NA	1934.9	NA
		No trend, with step	2060.0	Jul 2011 (Jun 2011***–***Jul 2011)	2011.1	Jul 2011 (Jun 2011***–***Aug 2011)	1943.5	Jul 2011 (Jun 2011***–***Aug 2011)
	Criteria C (Prior study)	***One change in trend, with step***	***2059.8***	***Jun 2011 (Feb 2010–Aug 2011)***	***2057.3***	***Jun 2011 (Mar 2011–Aug 2011)***	***2019.9***	***Jun 2011 (Mar 2011–Aug 2011)***
		One change in trend, no step	2061.7	Dec 2009 (Nov 2007***–***Aug 2010)	2059.6	Jan 2010 (Apr 2008***–***Sep 2010)	2022.2	Jan 2010 (Feb 2008***–***Oct 2010)
		Single overall trend	2186.4	NA	2178.6	NA	2130.0	NA
		No trend, with step	2207.2	Jun 2011 (Jun 2011***–***Aug 2011)	2167.8	Aug 2011 (Jun 2011***–***Dec 2011)	2108.6	Aug 2011 (Jun 2011***–***Dec 2011)
High-risk cases	Criteria A (Sensitivity/PPV)	One change in trend, with step	1553.0	May 2000 (Jan 2000***–***Feb 2009)	1553.2	May 2001 (Mar 2000***–***Feb 2009)	1516.1	May 2001 (Mar 2000***–***Feb 2009)
		***One change in trend, no step***	***1551.9***	***Sep 2001 (Aug 2000–May 2003)***	***1552.1***	***Sep 2001 (Sep 2000–Jul 2003)***	***1515.2***	***Oct 2001 (Nov 2000–Nov 2005)***
		Single overall trend	1579.8	NA	1583.7	NA	1552.4	NA
		No trend, with step	2141.6	Aug 2005 (Aug 2005***–***Nov 2007)	2019.9	Aug 2005 (Aug 2005***–***Aug 2005)	1854.1	Aug 2005 (Aug 2005***–***Aug 2005)
	Criteria B (Specificity/PPV)	***One change in trend, with step***	***1414.1***	***May 2015 (Mar 1999–Jan 2016)***	***1415.0***	***Jan 2000 (Mar 1999–Jan 2016)***	***1373.9***	***Jan 2000 (Jul 1999–Jul 2004)***
		One change in trend, no step	1417.5	Jun 2001 (Dec 1999***–***Mar 2016)	1417.5	Jun 2001 (Jan 2000***–***Jan 2016)	1376.1	Jun 2001 (Mar 2000***–***Aug 2005)
		Single overall trend	1422.6	NA	1423.8	NA	1384.6	NA
		No trend, with step	1903.7	May 2009 (Mar 2008***–***May 2009)	1836.6	Mar 2008 (Mar 2008***–***May 2009)	1708.5	Mar 2008 (Mar 2008***–***Mar 2008)
	Criteria C (Prior study)	***One change in trend, with step***	***1788.1***	***Jun 2002 (May 2002–Oct 2013)***	***1788.0***	***Jun 2002 (May 2002–Oct 2013)***	***1751.5***	***Jun 2002 (May 2002–Oct 2013)***
		One change in trend, no step	1791.9	Jun 2010 (Feb 2005***–***Jan 2012)	1791.9	Jun 2010 (Nov 2005***–***Feb 2012)	1756.8	Jun 2010 (Sep 2004***–***Nov 2012)
		Single overall trend	1804.0	NA	1802.9	NA	1764.4	NA
		No trend, with step	1939.9	Mar 2011 (Mar 2011***–***Aug 2011)	1920.5	Mar 2011 (Mar 2011***–***Dec 2011)	1874.2	Mar 2011 (Mar 2011***–***Aug 2011)

For the proportion of infective endocarditis cases with a streptococcal diagnosis code in HES (including mixtures) out of those with any organism coded, the best-fitting model for Criteria A was an upward step in October 2008 (IRR = 1.05 (1.02, 1.09), *p* < 0.01) with zero trend either side. For Criteria B, the best model was a downward step in June 2013 (IRR = 0.96 (0.92, 1.00), *p* = 0.07) with zero trend either side. For Criteria C, the best model was an upward trend (aIRR = 1.01 (01.00, 1.01), *p* = 0.06) until December 2012 where there was a downward step (IRR = 0.88 (0.81, 0.95), *p* < 0.001), after which there was another upward trend (aIRR = 1.03 (1.00, 1.05), *p* = 0.05) (Table 3).

**Table 3:** Variation in goodness of fit and optimal change point based on different models for the proportion of infective endocarditis cases associated with streptococcal organisms. For each model type, the month of change which gives the best model fit is shown, with best overall models shown in bold italics. AIC measures model goodness of fit (the lower the value the better the fit, within each set of inclusion criteria)

Inclusion criteria		Model type	AIC	Month of change (uncertainty interval)
Proportion of cases with a HES streptococcal code (including mixtures), out of all cases with *any* HES organism code	Criteria A (Sensitivity/PPV)	One change in trend, with step	1494.8	Oct 2008 (Dec 2005–Oct 2015)
		One change in trend, no step	1498.3	Apr 2002 (Oct 1997–Sep 2016)
		Single overall trend	1498.7	NA
		***No trend, with step***	***1493.4***	***Oct 2008 (Mar 2006***–***Apr 2010)***
	Criteria B (Specificity/PPV)	*One change in trend, with step*	*1415.1*	*Oct 2015 (Aug 2005*–*Dec 2015)*
		One change in trend, no step	1417.0	Sep 2011 (Oct 1997–Sep 2016)
		Single overall trend	1416.2	NA
		***No trend, with step***	***1413.3***	***Jun 2013 (Oct 1997***–***Sep 2016)***
	Criteria C (Prior study)	***One change in trend, with step***	***1508.4***	***Dec 2012 (Nov 2012***–***Oct 2013)***
		One change in trend, no step	1516.5	Dec 2010 (Oct 1997–Sep 2016)
		Single overall trend	1516.6	NA
		No trend, with step	1511.6	Dec 2012 (Apr 1999–May 2016)
Proportion of cases matched to an SGSS oral streptococcus sample (including mixtures), out of cases matched to *any* SGSS organism sample	Criteria A (Sensitivity/PPV)	One change in trend, with step	952.5	Mar 2008 (Mar 2007–Oct 2013)
		***One change in trend, no step***	***951.6***	***Dec 2008 (Jun 2007***–***Sep 2012)***
		Single overall trend	970.4	NA
		No trend, with step	960.1	Aug 2015 (Apr 2003–Oct 2015)
	Criteria B (Specificity/PPV)	One change in trend, with step	894.0	Sep 2013 (Mar 2006–Oct 2015)
		One change in trend, no step	892.9	May 2009 (Jan 2007–Feb 2015)
		Single overall trend	902.5	NA
		***No trend, with step***	***892.6***	***Aug 2015 (Sep 2013***–***Nov 2015)***
	Criteria C (Prior study)	One change in trend, with step	907.3	Oct 2013 (Mar 2007–Oct 2015)
		***One change in trend, no step***	***906.6***	***Jun 2012 (May 2007***–***Jun 2014)***
		Single overall trend	919.2	NA
		No trend, with step	907.0	Sep 2015 (Sep 2013–Nov 2015)

For the proportion of infective endocarditis cases matched to an oral *Streptococcus* record in SGSS (including mixtures) out of those with any organism identified in SGSS, the best-fitting model for Criteria A was an upward trend until December 2008 followed by a downward trend (aIRR = 1.07 (1.04, 1.11), *p* < 0.001 until December 2008, then aIRR = 0.98 (0.96, 0.99), *p* < 0.001 afterwards). For Criteria B, the best model was a downward step in August 2015 (IRR = 0.84 (0.77, 0.93), *p* < 0.001) with zero trend either side. For Criteria C, the best model was an upward trend until June 2012 followed by a downward trend (aIRR = 1.02 (1.00, 1.04), *p* = 0.03 until June 2012, then aIRR = 0.94 (0.92, 0.97), *p* < 0.001 afterwards) (Table 3).

**Fig. 5:**
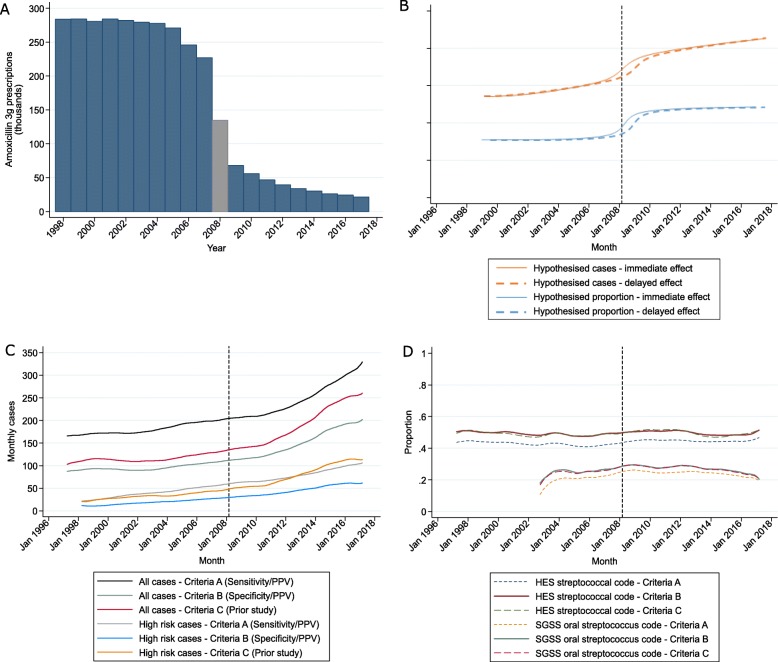
Temporal association of guideline change and incidence of infective endocarditis. **a** Annual prescriptions of 3 g amoxicillin dispensed in the community. Grey bar represents year of guideline change. **b** Hypothesised trend change in cases of infective endocarditis and in proportion of infective endocarditis cases linked to oral streptococci, assuming that dental antibiotic prophylaxis is protective of infective endocarditis (based on an assumed background of linearly increasing cases unrelated to oral prophylaxis). The solid line demonstrates an immediate effect, the dotted line demonstrates a delayed effect (assuming an incubation period of around 6 months—longer incubation periods would extend the delay in effect, and shorter incubation periods would move it closer to the solid line). **c** Actual trend change in cases of infective endocarditis after applying a non-parametric LOWESS smoother. **d** Actual trend change in proportion of infective endocarditis cases matched to a streptococcal organism code (including mixtures), after applying a non-parametric LOWESS smoother. Dotted vertical lines represent date of guideline change

Antibiotic prophylaxis prescribing dropped dramatically in 2008 (Fig. 5a). We hypothesised that if antibiotic prophylaxis were protective against the development of infective endocarditis, then both the incidence of infective endocarditis, and particularly cases associated with oral streptococci, would be a “mirror image” of the prescribing trend, though attenuated and with a possible delay in effect of 3–6 months (possible incubation period for infective endocarditis, longer lag periods would extend the period over which changes occurred, and shorter periods would reduce it) (Fig. 5b). There was no discernible change in the smoothed trends for overall and high-risk infective endocarditis cases in the time period around the guideline change in March 2008; incidence started increasing from 2010 (Fig. 5c). For the proportion of infective endocarditis cases associated with streptococcal organisms, again there was no apparent increase in the smoothed trends around the guideline change; the proportion with any streptococcal diagnosis code appeared constant over the entire period, while the proportion of oral streptococci appeared to increase gradually and then decrease, but with no clear “peak” date (Fig. 5d).

## Discussion

Cases of infective endocarditis are continuing to increase in England but this study found no evidence that there was any change in incidence associated specifically with the date of withdrawal of dental antibiotic prophylaxis as opposed to any other arbitrary date within the period of study. Controlling for population changes attenuated the increase in infective endocarditis but did not remove it. Statistical models suggested a wide variety of different “optimal” dates for a change in incidence trends, ranging from over 6.5 years before up to 7 years after the date of the guideline change. Models looking at the proportion of infective endocarditis cases associated with streptococcal species had optimal change points between 6 months and 7 years after the guideline change; however, the proportion of infective endocarditis cases associated specifically with oral streptococci actually *decreased* after the change points. While the optimal model for the proportion of Criteria A cases containing *any* streptococcal code from HES suggested an upward step in October 2008, there is no reason to believe that this one result is more informative than the other five results (including from the two other criteria) that suggested different dates and types of change point. Had there been a real change in incidence to detect, we would have expected there to be a clustering of results around a particular date and model, but this was not seen. There was also no discernible change in locally smoothed trends in infective endocarditis cases around the time the guidelines changed, nor any clear change in the proportion of infective endocarditis cases associated with oral streptococci. This was despite a clear and dramatic drop in antibiotic prescribing for dental prophylaxis.

When examining overall incidence trends, the choice of ICD-10 codes appeared to matter less than the strategy used for identifying incident cases. While the broad basket of codes used for Criteria A (maximising sensitivity and PPV) resulted in much higher estimates of incidence, the trend over time was very similar to that for Criteria B (which only used I330 primary codes and a similar strategy for identifying incident cases, maximising specificity and PPV). Contrastingly, there was a much steeper trend (both in all cases and in high-risk cases) for Criteria C post-2010 than there was for Criteria A or B. Since both Criteria B and C used the same ICD-10 codes, the difference in incidence trends is only explained by the choice of strategy for identifying incident cases (i.e. the exclusion of short stays, 30-day readmissions and all elective admissions for Criteria B, versus the exclusion of “Elective – waiting list” admissions for Criteria C). Explicit exclusion of readmissions is particularly important as efforts to reduce length of stay in English hospitals over the last decade have seen concurrent increases in readmissions. Alternatively, some attendances for Outpatient Parental Antibiotic Therapy, increasingly used to provide long intravenous antibiotic courses, may have been incorrectly coded as inpatient admissions, artificially inflating case numbers.

The main strength of this study is the inclusion of microbiological data from SGSS that distinguishes oral streptococci from other streptococcal species. Although the proportion of infective endocarditis cases that could be matched to a microbiological sample was typically below 50% and changed considerably over time, the agreement between the organisms found in SGSS versus HES was regularly around 90%, suggesting that when organism codes are present, they are probably reliable. (The increase in numbers matched likely reflects additional microbiology laboratories joining SGSS, reducing variability in estimated agreement over time.) Despite this, the organisms isolated from a patient with an infective endocarditis episode cannot be guaranteed to be the cause of the infective endocarditis episode as opposed to another co-occurring infection or blood culture contaminant, which could explain some of the discrepancies.

One limitation is that, while the numbers of procedures for prosthetic valve replacement and repair have undoubtedly increased over the last decade, we did not have access to mortality data and only had HES episodes that contained endocarditis codes, so were not able to attempt to estimate how much of the increase could be explained simply by an increase in the high-risk population. However, the upward trend in infective endocarditis incidence is clearly not limited to this group, as it was still visible in the population with no recorded history of these procedures (Additional file 2: Figure S2A). Similarly, as in previous studies, we did not have access to data on the population actually undergoing invasive dental procedures to use as a denominator; we attempted to assess the potentially dental-exposed population by looking at cases of infective endocarditis associated with organisms that are known to reside in the mouth (in particular oral streptococci) and did not find any increase in these cases.

A further strength of the study is the variety of statistical methods used, which showed how interpretation can be influenced by choice of model and/or coding criteria. However, another limitation is that our models only allowed for at most one change in trend, albeit with and without an additional increase in incidence, and were restricted to log-linear associations with time. While in theory other, more complex models might have fitted the data better, non-parametric smoothed trends suggest our modelling strategy was not unreasonable. Since our identification of high-risk cases was dependent on coding in earlier years, it is possible that cases in earlier periods (where there were fewer years of previous codes available) are underestimated compared to cases in later years. However, this would create a bias towards finding an increase in incidence after the guideline change and therefore does not affect our conclusion that increases could not be specifically linked to timing of guideline change. Another more general limitation is that since data from EHRs such as HES are collected principally for administrative reasons rather than for research, they are potentially subject to (and biased by) operational factors that we may simply not be aware of.

The most recent comparable study using English data was published in 2015 [6] and reported an increase in the incidence rate of infective endocarditis following publication of the NICE guidelines. However, when we used different case definitions based on a recent study [17] and different statistical methods which identify the most likely date that trends changed, we found a wide range of likely dates for a change in incidence trends, leading us to conclude that there is no evidence for a direct link with the change in guidance in 2008. Although Criteria C implements the inclusion as reported in the earlier study [6], we found small differences in estimated incidence compared to this publication and found much higher and more stable coverage of secondary ICD-10 codes for organisms than previously reported, despite theoretically using the same underlying HES data. Studies from other countries have reported varying results, some seeing overall increases in infective endocarditis [10–13] and some not [4, 5, 7–9, 14, 15], though those that we are aware of which looked specifically at cases associated with oral streptococci did not report an increase after guideline changes [7, 8, 15]. It is of course still possible that there is an increased risk of developing infective endocarditis after an invasive dental procedure [24], but the vast majority of cases appear to be unrelated to such procedures, and the efficacy of antibiotic prophylaxis in preventing cases is still inconclusive.

## Conclusions

We find no evidence that the change in guidelines for dental antibiotic prophylaxis has increased the incidence of infective endocarditis in England, since neither the trends in incident cases nor in the proportion of cases associated with oral streptococci (i.e. cases more likely to be associated with invasive dental procedures) appeared to correspond to the clear change in dental antibiotic prescribing. Statistical tests for changes in trend were highly statistically significant across a wide range of time points, but the optimal time of change identified was sensitive to differences in inclusion criteria and choice of model. Focussing on evidence for changes after vs before a single time point in one outcome with one analysis method may be problematic in large ecological studies of this type. Non-parametric smoothing can be used as a helpful “sense check”.

Large observational studies based on EHRs are becoming increasingly common and are attractive given their high power and relatively low cost. However, such studies need to be conducted very carefully, including the use of extensive sensitivity analyses as demonstrated here, because their higher power makes the finding of statistically significant results much more likely. Although we find no evidence that the withdrawal of dental antibiotic prophylaxis has increased cases of infective endocarditis, we do find that infective endocarditis has continued to increase rapidly in England, with incidence roughly doubling over the 20 years of the study. Further research should focus on determining the true cause of this increase.

## Supplementary information

Additional file 1: Supplementary tables. All supplementary tables to accompany the manuscript. **Table S1.** Diagnosis (ICD-10) and procedure (OPCS-4) codes identifying high-risk individuals with pre-existing prosthetic valve or congenital heart disease. **Table S2.** Secondary ICD-10 codes for potential causal organisms. **Table S3.** Classification of SGSS organisms into subgroups. **Table S4.** Results of an interrupted time-series analysis testing for a difference in trend before and after a fixed date, for each month in the study period using Poisson regression, for Criteria A. (PDF 753 kb)

Additional file 2: Supplementary figures. All supplementary figures to accompany the manuscript. **Figure S1.** Effect of applying different methods to adjust for changes in population, for all 3 criteria. **Figure S2.** Monthly cases of infective endocarditis excluding individuals identified as high-risk or as illicit drug users. **Figure S3.** Causative organism based on secondary diagnosis codes in HES, for all 3 criteria. **Figure S4.** Of infective endocarditis cases with an organism code present in HES, proportion that were coded as streptococcal, staphylococcal, or other/unnamed (including mixtures), for all 3 criteria. **Figure S5.** Of infective endocarditis cases with an organism code present in HES, proportion that were coded exclusively as streptococcal, staphylococcal or other/unnamed, or else with a mixture of codes, for all 3 criteria. **Figure S6.** Causative organism based on SGSS: monthly agreement of SGSS organism compared to HES organism code, based on 3 groups: streptococcal, staphylococcal, other/unnamed, for all 3 criteria. **Figure S7.** Of all infective endocarditis cases that were matched to an organism in SGSS, proportion that were classed as oral streptococci, for all 3 criteria. **Figure S8**. Infective endocarditis cases that were matched to an organism in SGSS. The HACEK group consists of Haemophilus species, Aggregatibacter (previously Actinobacillus), Cardiobacterium, Eikenella, Kingella. CONS – Coagulase negative staphylococci. (PDF 231 kb)

## Abbreviations

AHA: American Heart Association
AIC: Akaike Information Criterion
aIRR: Annual incidence rate ratio
EHRs: Electronic health records
ESC: European Society of Cardiology
HES: Hospital Episode Statistics
ICD-10: International Classification of Diseases, Tenth Revision
IRR: Incidence rate ratio
NHS: National Health Service
NICE: National Institute for Health and Care Excellence
PPV: Positive predictive value
SGSS: Second Generation Surveillance System - Public Health England’s centralised collection of microbiology results from English laboratories
