# Supplementary material for: Investigation of the impact of the NICE guidelines regarding antibiotic prophylaxis during invasive dental procedures on the incidence of infective endocarditis in England: an electronic health records study
Source: BMC Med. 2020 Apr 2;18:84. doi: 10.1186/s12916-020-01531-y (PMC7114779; doi:10.1186/s12916-020-01531-y)
Supplement: Supplementary file 1 — Supplementary tables. All supplementary tables to accompany the manuscript. Table S1. Diagnosis (ICD-10) and procedure (OPCS-4) codes identifying high-risk individuals with pre-existing prosthetic valve or congenital heart disease. Table S2. Secondary ICD-10 codes for potential causal organisms. Table S3. Classification of SGSS organisms into subgroups. Table S4. Results of an interrupted time-series analysis testing for a difference in trend before and after a fixed date, for each month in the study period using Poisson regression, for Criteria A. (PDF 753 kb) [file 12916_2020_1531_MOESM1_ESM.pdf]

Investigation of the impact of the NICE guidelines regarding antibiotic prophylaxis during invasive dental procedures on the incidence of infective endocarditis in England: an Electronic Health Records study - Supplementary tables

Table S1. Diagnosis (ICD-10) and procedure (OPCS-4) codes identifying high-risk individuals with pre-existing prosthetic valve or congenital heart disease, as defined by Dayer *et al*. Patients were categorised as high-risk if they had any previous admissions that included one of the codes in the table, except for codes representing congenital heart disease repaired with prosthetic material, which were only relevant if they occurred in the 6 months preceding the admission with endocarditis. Patients with a prior history of endocarditis are also considered high risk, and any previous admissions for infective endocarditis were determined using the same case definitions for each of Criteria A, B or C.

**OPCS-4 codes for prosthetic replacement of a heart valve:**
K25.1-K25.4, K26.1-K26.4, K27.1-K27-4, K28.1-K28.4 or K29.1-K29.4
**OPCS-4 codes for valve repair using prosthetic material:**
K25.5, K25.8, K25.9, K26.8, K26.9, K27.6, K27.8, K27.9, K28.8, K28.9, K29.8, K29.9, K30.1-K30.5, K30.8, K30.9, K33.1-K33.9, K34.1-K34.3, K35.8+Z32.4.
**OPCS-4 codes for congenital heart disease repaired with prosthetic material:**
K09.1, K09.2, K10.1, K11.1, K11.7, K12.1, K13.1-K13.5, K13.8, K13.9, K16.3, K16.5, L03.1, L07.5, L10.1, L23.3
**OPCS-4 codes for congenital heart disease patients in whom a palliative shunt or conduit had been used:**
K04.1, K04.2, K06.3, K17.1, K17.3, K17.4, K18.1-K19.9, K76.1, K76.8, K76.9, L05.1-L05.3, L05.8, L05.9, L07.1-L07.3, L07.8, L07.9
**ICD-10 codes for a pre-existing congenital cyanotic heart condition:**
Q20.0-Q20.4, Q21.2-Q21.8, Q26.2
**OPCS-4 codes for prosthetic heart/ventricular assist device:**
K02.3, K02.5, K54.1

Table S2. Secondary ICD-10 codes for potential causal organisms

ICD10 code	ICD10 description	Overall group
A400	Sepsis due to streptococcus, group A	Streptococcus
A401	Sepsis due to streptococcus, group B	Streptococcus
A402	Sepsis due to streptococcus, group D	Streptococcus
A403	Sepsis due to Streptococcus pneumoniae	Streptococcus
A408	Other streptococcal sepsis	Streptococcus
A409	Streptococcal sepsis, unspecified	Streptococcus
A491	Streptococcal and enterococcal infection, unspecified site	Streptococcus
B950	Streptococcus, group A, as the cause of diseases classified to other chapters	Streptococcus
B951	Streptococcus, group B, as the cause of diseases classified to other chapters	Streptococcus
B952	Streptococcus group D and enterococcus as the cause of diseases classified to other chapters	Streptococcus
B953	Streptococcus pneumoniae as the cause of diseases classified to other chapters	Streptococcus
B954	Other streptococcus as the cause of diseases classified to other chapters	Streptococcus
B955	Unspecified streptococcus as the cause of diseases classified to other chapters	Streptococcus
A410	Sepsis due to Staphylococcus aureus	Staphylococcus
A411	Sepsis due to other specified staphylococcus	Staphylococcus

ICD10 code	ICD10 description	Overall group
A412	Sepsis due to unspecified staphylococcus	Staphylococcus
A490	Staphylococcal infection, unspecified site	Staphylococcus
B956	Staphylococcus aureus as the cause of diseases classified to other chapters	Staphylococcus
B957	Other staphylococcus as the cause of diseases classified to other chapters	Staphylococcus
B958	Unspecified staphylococcus as the cause of diseases classified to other chapters	Staphylococcus
A413	Sepsis due to Haemophilus influenzae	Other
A414	Sepsis due to anaerobes	Other
A415	Sepsis due to other Gram-negative organisms	Other
A418	Other specified sepsis	Other
A492	Haemophilus influenzae infection, unspecified site	Other
A493	Mycoplasma infection, unspecified site	Other
A498	Other bacterial infections of unspecified site	Other
A499	Bacterial infection, unspecified	Other
B96	Other specified bacterial agents as the cause of diseases classified to other chapters	Other
B960	Mycoplasma pneumoniae [M. pneumoniae] as the cause of diseases classified to other chapters	Other
B961	Klebsiella pneumoniae [K. pneumoniae] as the cause of diseases classified to other chapters	Other
B962	Escherichia coli [E. coli] as the cause of diseases classified to other chapters	Other
B963	Haemophilus influenzae [H. influenzae] as the cause of diseases classified to other chapters	Other
B964	Proteus (mirabilis)(morganii) as the cause of diseases classified to other chapters	Other
B965	Pseudomonas (aeruginosa) as the cause of diseases classified to other chapters	Other
B966	Bacillus fragilis [B. fragilis] as the cause of diseases classified to other chapters	Other
B967	Clostridium perfringens [C. perfringens] as the cause of diseases classified to other chapters	Other
B968	Other specified bacterial agents as the cause of diseases classified to other chapters	Other
B97	Viral agents as the cause of diseases classified to other chapters	Other
B970	Adenovirus as the cause of diseases classified to other chapters	Other
B971	Enterovirus as the cause of diseases classified to other chapters	Other
B972	Coronavirus as the cause of diseases classified to other chapters	Other
B973	Retrovirus as the cause of diseases classified to other chapters	Other
B974	Respiratory syncytial virus as the cause of diseases classified to other chapters	Other
B975	Reovirus as the cause of diseases classified to other chapters	Other
B976	Parvovirus as the cause of diseases classified to other chapters	Other
B977	Papillomavirus as the cause of diseases classified to other chapters	Other
B978	Other viral agents as the cause of diseases classified to other chapters	Other
B98	Other specified infectious agents as the cause of diseases classified to other chapters	Other
B980	Helicobacter pylori [H.pylori] as the cause of diseases classified to other chapters	Other
B981	Vibrio vulnificus as the cause of diseases classified to other chapters	Other
B99	Other and unspecified infectious diseases	Other

Table S3. Classification of SGSS organisms into subgroups

**Organism name**	**Overall group**	**Subgroup**
ALPHA HAEMOLYTIC STREPTOCOCCI	Streptococcus species	Oral streptococci
STREPTOCOCCI SALIVARIUS	Streptococcus species	Oral streptococci
STREPTOCOCCUS ACIDOMINIMUS	Streptococcus species	Oral streptococci
STREPTOCOCCUS CONSTELLATUS (MILLERI GROUP)	Streptococcus species	Oral streptococci
STREPTOCOCCUS GORDONII	Streptococcus species	Oral streptococci
STREPTOCOCCUS MILLERI	Streptococcus species	Oral streptococci
STREPTOCOCCUS MITIS	Streptococcus species	Oral streptococci
STREPTOCOCCUS MUTANS	Streptococcus species	Oral streptococci
STREPTOCOCCUS ORALIS	Streptococcus species	Oral streptococci
STREPTOCOCCUS PNEUMONIAE	Streptococcus species	Oral streptococci
STREPTOCOCCUS SALIVARIUS	Streptococcus species	Oral streptococci
STREPTOCOCCUS SANGUINIS	Streptococcus species	Oral streptococci
STREPTOCOCCUS SANGUIS	Streptococcus species	Oral streptococci
STREPTOCOCCUS SANGUIS II	Streptococcus species	Oral streptococci
STREPTOCOCCUS VESTIBULARIS	Streptococcus species	Oral streptococci
STREPTOCOCCUS VIRIDANS	Streptococcus species	Oral streptococci
STREPTOCOCCUS VIRIDANS SPECIES	Streptococcus species	Oral streptococci
GROUP A STREPTOCOCCI	Streptococcus species	Pyogenic streptococci
STREPTOCOCCI, BETA HAEMOLYTIC GROUP B	Streptococcus species	Pyogenic streptococci
STREPTOCOCCI, BETA HAEMOLYTIC GROUP C	Streptococcus species	Pyogenic streptococci
STREPTOCOCCI, BETA HAEMOLYTIC GROUP G	Streptococcus species	Pyogenic streptococci
STREPTOCOCCUS EQUISIMILIS	Streptococcus species	Pyogenic streptococci
STREPTOCOCCUS PYOGENES SERO GROUP A	Streptococcus species	Pyogenic streptococci
STREPTOCOCCUS BOVIS	Streptococcus species	Group D streptococci
STREPTOCOCCUS GALLOLYTICUS (Strep.bovis biotype I)	Streptococcus species	Group D streptococci
ABIOTROPHIA ADIACENS	Streptococcus species	Other streptococci
GEMELLA HAEMOLYSANS	Streptococcus species	Other streptococci
GEMELLA MORBILLORUM	Streptococcus species	Other streptococci
GEMELLA SPECIES	Streptococcus species	Other streptococci
LACTOCOCCUS LACTIS	Streptococcus species	Other streptococci
STREPTOCOCCI	Streptococcus species	Other streptococci
STREPTOCOCCUS ADJACENS	Streptococcus species	Other streptococci
METHICILLIN RESISTANT STAPHYLOCOCCUS AUREUS (MRSA)	Staphylococcus species	Staphylococcus aureus
METHICILLIN RESISTANT STAPHYLOCOCCUS AUREUS (MRSA) ISOLATED	Staphylococcus species	Staphylococcus aureus
STAPHYLOCOCCUS AUREUS	Staphylococcus species	Staphylococcus aureus
COAGULASE NEGATIVE STAPHYLOCOCCUS	Staphylococcus species	Coagulase negative staphylococci
COAGULASE NEGATIVE STAPHYLOCOCCUS 1	Staphylococcus species	Coagulase negative staphylococci
COAGULASE NEGATIVE STAPHYLOCOCCUS 2	Staphylococcus species	Coagulase negative staphylococci

**Organism name**	**Overall group**	**Subgroup**
COAGULASE NEGATIVE STAPHYLOCOCCUS 3	Staphylococcus species	Coagulase negative staphylococci
STAPHYLOCOCCUS EPIDERMIDIS(CoNS)	Staphylococcus species	Coagulase negative staphylococci
STAPHYLOCOCCUS HOMINIS	Staphylococcus species	Coagulase negative staphylococci
STAPHYLOCOCCUS LUGDUNENSIS(CoNS)	Staphylococcus species	Coagulase negative staphylococci
STAPHYLOCOCCUS WARNERI	Staphylococcus species	Coagulase negative staphylococci
(ACTINOBACILLUS ACTINOMYCETEMCOMITANS) HAEMOPHILUS ACTINOMYCETEMCOMITANS	Other	Other, HACEK
ACTINOBACILLUS ACTINOMYCETEMCOMITANS	Other	Other, HACEK
CARDIOBACTERIUM HOMINIS	Other	Other, HACEK
HAEMOPHILUS APHROPHILUS	Other	Other, HACEK
HAEMOPHILUS INFLUENZAE	Other	Other, HACEK
HAEMOPHILUS PARAINFLUENZAE	Other	Other, HACEK
KINGELLA KINGAE	Other	Other, HACEK
ENTEROCOCCI	Other	Enterococci
ENTEROCOCCUS DURANS GROUP D	Other	Enterococci
ENTEROCOCCUS FAECALIS GROUP D	Other	Enterococci
ENTEROCOCCUS FAECIUM (VRE)	Other	Enterococci
ENTEROCOCCUS FAECIUM GROUP D	Other	Enterococci
ENTEROCOCCUS GALLINARUM (VRE)	Other	Enterococci
ENTEROCOCCUS GALLINARUM GROUP D	Other	Enterococci
ENTEROCOCCUS SPECIES	Other	Enterococci
VANCOMYCIN RESISTANT ENTEROCOCCUS	Other	Enterococci
ACINETOBACTER SPECIES	Other	Other
AEROBIC SPORE BEARER	Other	Other
AEROCOCCUS URINAE	Other	Other
ARCANOBACTERIUM BERNARDIAE	Other	Other
CANDIDA	Other	Other
CANDIDA ALBICANS	Other	Other
CANDIDA GLABRATA	Other	Other
CANDIDA KRUSEI	Other	Other
CANDIDA PARAPSILOSIS	Other	Other
CANDIDA SPECIES	Other	Other
CAPNOCYTOPHAGA CANIMORSUS	Other	Other
CAPNOCYTOPHAGA SPECIES	Other	Other
CITROBACTER FREUNDII	Other	Other
CORYNEBACTERIUM AFERMENTANS	Other	Other
CORYNEBACTERIUM JEIKEIUM	Other	Other
CORYNEBACTERIUM PROPINQUUM	Other	Other
CORYNEBACTERIUM SPECIES	Other	Other
CORYNEBACTERIUM STRIATUM	Other	Other
DIPHTHEROIDS	Other	Other

**Organism name**	**Overall group**	**Subgroup**
ENTEROBACTER CLOACAE	Other	Other
ESCHERICHIA COLI	Other	Other
ESCHERICHIA COLI(ESBL)	Other	Other
FLAVIMONAS ORYZIHABITANS	Other	Other
KLEBSIELLA OXYTOCA	Other	Other
KLEBSIELLA PNEUMONIAE	Other	Other
LACTOBACILLUS CASEI	Other	Other
LACTOBACILLUS RHAMNOSUS	Other	Other
LACTOBACILLUS SPECIES	Other	Other
MICROCOCCUS SPECIES	Other	Other
NEISSERIA CINEREA	Other	Other
PANTOEA SPECIES	Other	Other
PROPIONIBACTERIUM SPECIES	Other	Other
PROTEUS MIRABILIS	Other	Other
PROTEUS SPECIES	Other	Other
PSEUDOMONAS AERUGINOSA	Other	Other
RALSTONIA PICKETTII	Other	Other
SERRATIA MARCESCENS	Other	Other
STENOTROPHOMONAS MALTOPHILIA	Other	Other

Table S4: Results of an interrupted time-series analysis testing for a difference in trend before and after a fixed date, for each month in the study period using negative binomial regression (as there was evidence of overdispersion), for Criteria A. As can be seen in the table, only 8 of the 238 dates tested did not show a significant increase in trend after vs before this date, all of which were at the extreme ends of the study period and were a consequence of wide confidence intervals due to the small number of data points at the extreme ends, as opposed to the point estimates being closer. Results were similar for Criteria B and C.

**Month of change**	**Trend in first period (annual IRR (95%CI))**	**Trend in second period (annual IRR (95%CI))**	**p-value for difference in trends**
1997m4	-	1.03 (1.03, 1.03)	-
1997m5	0.10 (0.01, 1.11)	1.03 (1.03, 1.03)	0.057
1997m6	0.49 (0.16, 1.48)	1.03 (1.03, 1.03)	0.184
1997m7	0.69 (0.35, 1.36)	1.03 (1.03, 1.03)	0.247
1997m8	0.76 (0.48, 1.21)	1.03 (1.03, 1.03)	0.193
1997m9	0.81 (0.57, 1.14)	1.03 (1.03, 1.03)	0.161
1997m10	0.83 (0.63, 1.08)	1.03 (1.03, 1.03)	0.110
1997m11	0.83 (0.67, 1.04)	1.03 (1.03, 1.03)	0.058
1997m12	0.85 (0.71, 1.02)	1.03 (1.03, 1.03)	0.036
1998m1	0.86 (0.74, 1.01)	1.03 (1.03, 1.03)	0.025
1998m2	0.88 (0.77, 1.00)	1.03 (1.03, 1.04)	0.017
1998m3	0.89 (0.79, 1.00)	1.03 (1.03, 1.04)	0.012
1998m4	0.90 (0.81, 1.00)	1.03 (1.03, 1.04)	0.008
1998m5	0.91 (0.83, 0.99)	1.03 (1.03, 1.04)	0.006
1998m6	0.91 (0.84, 0.99)	1.03 (1.03, 1.04)	0.004
1998m7	0.92 (0.85, 0.99)	1.03 (1.03, 1.04)	0.003
1998m8	0.93 (0.86, 0.99)	1.03 (1.03, 1.04)	0.002
1998m9	0.93 (0.87, 0.99)	1.03 (1.03, 1.04)	0.002
1998m10	0.94 (0.88, 0.99)	1.03 (1.03, 1.04)	0.001
1998m11	0.94 (0.89, 0.99)	1.03 (1.03, 1.04)	<0.001
1998m12	0.94 (0.90, 0.99)	1.03 (1.03, 1.04)	<0.001
1999m1	0.95 (0.90, 0.99)	1.03 (1.03, 1.04)	<0.001
1999m2	0.95 (0.91, 1.00)	1.03 (1.03, 1.04)	<0.001
1999m3	0.96 (0.92, 1.00)	1.03 (1.03, 1.04)	<0.001
1999m4	0.96 (0.92, 1.00)	1.03 (1.03, 1.04)	<0.001
1999m5	0.96 (0.93, 1.00)	1.03 (1.03, 1.04)	<0.001
1999m6	0.96 (0.93, 1.00)	1.03 (1.03, 1.04)	<0.001
1999m7	0.97 (0.93, 1.00)	1.03 (1.03, 1.04)	<0.001
1999m8	0.97 (0.94, 1.00)	1.03 (1.03, 1.04)	<0.001
1999m9	0.97 (0.94, 1.00)	1.03 (1.03, 1.04)	<0.001
1999m10	0.97 (0.94, 1.00)	1.03 (1.03, 1.04)	<0.001
1999m11	0.97 (0.94, 1.00)	1.03 (1.03, 1.04)	<0.001
1999m12	0.97 (0.95, 1.00)	1.03 (1.03, 1.04)	<0.001
2000m1	0.97 (0.95, 1.00)	1.04 (1.03, 1.04)	<0.001
2000m2	0.97 (0.95, 1.00)	1.04 (1.03, 1.04)	<0.001
2000m3	0.98 (0.95, 1.00)	1.04 (1.03, 1.04)	<0.001
2000m4	0.98 (0.95, 1.00)	1.04 (1.03, 1.04)	<0.001
2000m5	0.98 (0.96, 1.00)	1.04 (1.03, 1.04)	<0.001
2000m6	0.98 (0.96, 1.00)	1.04 (1.03, 1.04)	<0.001
2000m7	0.98 (0.96, 1.00)	1.04 (1.03, 1.04)	<0.001

Month of change	Trend in first period (annual IRR (95%CI))	Trend in second period (annual IRR (95%CI))	p-value for difference in trends
2000m8	0.98 (0.96, 1.00)	1.04 (1.03, 1.04)	<0.001
2000m9	0.98 (0.96, 1.00)	1.04 (1.03, 1.04)	<0.001
2000m10	0.98 (0.96, 1.00)	1.04 (1.03, 1.04)	<0.001
2000m11	0.98 (0.97, 1.00)	1.04 (1.03, 1.04)	<0.001
2000m12	0.98 (0.97, 1.00)	1.04 (1.03, 1.04)	<0.001
2001m1	0.99 (0.97, 1.00)	1.04 (1.03, 1.04)	<0.001
2001m2	0.99 (0.97, 1.00)	1.04 (1.03, 1.04)	<0.001
2001m3	0.99 (0.97, 1.00)	1.04 (1.03, 1.04)	<0.001
2001m4	0.99 (0.97, 1.00)	1.04 (1.03, 1.04)	<0.001
2001m5	0.99 (0.97, 1.00)	1.04 (1.03, 1.04)	<0.001
2001m6	0.99 (0.98, 1.00)	1.04 (1.03, 1.04)	<0.001
2001m7	0.99 (0.98, 1.00)	1.04 (1.03, 1.04)	<0.001
2001m8	0.99 (0.98, 1.00)	1.04 (1.03, 1.04)	<0.001
2001m9	0.99 (0.98, 1.01)	1.04 (1.03, 1.04)	<0.001
2001m10	0.99 (0.98, 1.01)	1.04 (1.03, 1.04)	<0.001
2001m11	0.99 (0.98, 1.01)	1.04 (1.03, 1.04)	<0.001
2001m12	0.99 (0.98, 1.01)	1.04 (1.03, 1.04)	<0.001
2002m1	0.99 (0.98, 1.01)	1.04 (1.04, 1.04)	<0.001
2002m2	1.00 (0.98, 1.01)	1.04 (1.04, 1.04)	<0.001
2002m3	1.00 (0.98, 1.01)	1.04 (1.04, 1.04)	<0.001
2002m4	1.00 (0.99, 1.01)	1.04 (1.04, 1.04)	<0.001
2002m5	1.00 (0.99, 1.01)	1.04 (1.04, 1.04)	<0.001
2002m6	1.00 (0.99, 1.01)	1.04 (1.04, 1.04)	<0.001
2002m7	1.00 (0.99, 1.01)	1.04 (1.04, 1.04)	<0.001
2002m8	1.00 (0.99, 1.01)	1.04 (1.04, 1.04)	<0.001
2002m9	1.00 (0.99, 1.01)	1.04 (1.04, 1.04)	<0.001
2002m10	1.00 (0.99, 1.01)	1.04 (1.04, 1.04)	<0.001
2002m11	1.00 (0.99, 1.01)	1.04 (1.04, 1.04)	<0.001
2002m12	1.00 (0.99, 1.01)	1.04 (1.04, 1.04)	<0.001
2003m1	1.00 (0.99, 1.01)	1.04 (1.04, 1.04)	<0.001
2003m2	1.00 (0.99, 1.01)	1.04 (1.04, 1.04)	<0.001
2003m3	1.00 (0.99, 1.01)	1.04 (1.04, 1.04)	<0.001
2003m4	1.00 (0.99, 1.01)	1.04 (1.04, 1.04)	<0.001
2003m5	1.00 (0.99, 1.01)	1.04 (1.04, 1.04)	<0.001
2003m6	1.00 (1.00, 1.01)	1.04 (1.04, 1.04)	<0.001
2003m7	1.00 (1.00, 1.01)	1.04 (1.04, 1.04)	<0.001
2003m8	1.00 (1.00, 1.01)	1.04 (1.04, 1.04)	<0.001
2003m9	1.00 (1.00, 1.01)	1.04 (1.04, 1.04)	<0.001
2003m10	1.01 (1.00, 1.01)	1.04 (1.04, 1.04)	<0.001
2003m11	1.01 (1.00, 1.01)	1.04 (1.04, 1.04)	<0.001
2003m12	1.01 (1.00, 1.01)	1.04 (1.04, 1.04)	<0.001
2004m1	1.01 (1.00, 1.01)	1.04 (1.04, 1.04)	<0.001
2004m2	1.01 (1.00, 1.01)	1.04 (1.04, 1.04)	<0.001
2004m3	1.01 (1.00, 1.01)	1.04 (1.04, 1.04)	<0.001
2004m4	1.01 (1.00, 1.01)	1.04 (1.04, 1.04)	<0.001
2004m5	1.01 (1.00, 1.01)	1.04 (1.04, 1.04)	<0.001
2004m6	1.01 (1.00, 1.02)	1.04 (1.04, 1.04)	<0.001
2004m7	1.01 (1.00, 1.02)	1.04 (1.04, 1.04)	<0.001

Month of change	Trend in first period (annual IRR (95%CI))	Trend in second period (annual IRR (95%CI))	p-value for difference in trends
2004m8	1.01 (1.00, 1.02)	1.04 (1.04, 1.05)	<0.001
2004m9	1.01 (1.00, 1.02)	1.04 (1.04, 1.05)	<0.001
2004m10	1.01 (1.00, 1.02)	1.04 (1.04, 1.05)	<0.001
2004m11	1.01 (1.00, 1.02)	1.04 (1.04, 1.05)	<0.001
2004m12	1.01 (1.00, 1.02)	1.04 (1.04, 1.05)	<0.001
2005m1	1.01 (1.00, 1.02)	1.04 (1.04, 1.05)	<0.001
2005m2	1.01 (1.00, 1.02)	1.04 (1.04, 1.05)	<0.001
2005m3	1.01 (1.00, 1.02)	1.04 (1.04, 1.05)	<0.001
2005m4	1.01 (1.00, 1.02)	1.04 (1.04, 1.05)	<0.001
2005m5	1.01 (1.00, 1.02)	1.04 (1.04, 1.05)	<0.001
2005m6	1.01 (1.01, 1.02)	1.04 (1.04, 1.05)	<0.001
2005m7	1.01 (1.01, 1.02)	1.04 (1.04, 1.05)	<0.001
2005m8	1.01 (1.01, 1.02)	1.04 (1.04, 1.05)	<0.001
2005m9	1.01 (1.01, 1.02)	1.04 (1.04, 1.05)	<0.001
2005m10	1.01 (1.01, 1.02)	1.04 (1.04, 1.05)	<0.001
2005m11	1.01 (1.01, 1.02)	1.04 (1.04, 1.05)	<0.001
2005m12	1.01 (1.01, 1.02)	1.04 (1.04, 1.05)	<0.001
2006m1	1.01 (1.01, 1.02)	1.04 (1.04, 1.05)	<0.001
2006m2	1.01 (1.01, 1.02)	1.04 (1.04, 1.05)	<0.001
2006m3	1.01 (1.01, 1.02)	1.04 (1.04, 1.05)	<0.001
2006m4	1.01 (1.01, 1.02)	1.05 (1.04, 1.05)	<0.001
2006m5	1.01 (1.01, 1.02)	1.05 (1.04, 1.05)	<0.001
2006m6	1.01 (1.01, 1.02)	1.05 (1.04, 1.05)	<0.001
2006m7	1.01 (1.01, 1.02)	1.05 (1.04, 1.05)	<0.001
2006m8	1.01 (1.01, 1.02)	1.05 (1.04, 1.05)	<0.001
2006m9	1.01 (1.01, 1.02)	1.05 (1.04, 1.05)	<0.001
2006m10	1.01 (1.01, 1.02)	1.05 (1.04, 1.05)	<0.001
2006m11	1.01 (1.01, 1.02)	1.05 (1.04, 1.05)	<0.001
2006m12	1.01 (1.01, 1.02)	1.05 (1.04, 1.05)	<0.001
2007m1	1.01 (1.01, 1.02)	1.05 (1.04, 1.05)	<0.001
2007m2	1.01 (1.01, 1.02)	1.05 (1.04, 1.05)	<0.001
2007m3	1.01 (1.01, 1.02)	1.05 (1.04, 1.05)	<0.001
2007m4	1.01 (1.01, 1.02)	1.05 (1.04, 1.05)	<0.001
2007m5	1.01 (1.01, 1.02)	1.05 (1.04, 1.05)	<0.001
2007m6	1.01 (1.01, 1.02)	1.05 (1.04, 1.05)	<0.001
2007m7	1.02 (1.01, 1.02)	1.05 (1.04, 1.05)	<0.001
2007m8	1.02 (1.01, 1.02)	1.05 (1.04, 1.05)	<0.001
2007m9	1.02 (1.01, 1.02)	1.05 (1.05, 1.05)	<0.001
2007m10	1.02 (1.01, 1.02)	1.05 (1.05, 1.05)	<0.001
2007m11	1.02 (1.01, 1.02)	1.05 (1.05, 1.06)	<0.001
2007m12	1.02 (1.01, 1.02)	1.05 (1.05, 1.06)	<0.001
2008m1	1.02 (1.01, 1.02)	1.05 (1.05, 1.06)	<0.001
2008m2	1.02 (1.01, 1.02)	1.05 (1.05, 1.06)	<0.001
2008m3	1.02 (1.01, 1.02)	1.05 (1.05, 1.06)	<0.001
2008m4	1.02 (1.01, 1.02)	1.05 (1.05, 1.06)	<0.001
2008m5	1.02 (1.01, 1.02)	1.05 (1.05, 1.06)	<0.001
2008m6	1.02 (1.01, 1.02)	1.05 (1.05, 1.06)	<0.001
2008m7	1.02 (1.01, 1.02)	1.05 (1.05, 1.06)	<0.001

Month of change	Trend in first period (annual IRR (95%CI))	Trend in second period (annual IRR (95%CI))	p-value for difference in trends
2008m8	1.02 (1.01, 1.02)	1.05 (1.05, 1.06)	<0.001
2008m9	1.02 (1.01, 1.02)	1.05 (1.05, 1.06)	<0.001
2008m10	1.02 (1.01, 1.02)	1.05 (1.05, 1.06)	<0.001
2008m11	1.02 (1.01, 1.02)	1.05 (1.05, 1.06)	<0.001
2008m12	1.02 (1.01, 1.02)	1.06 (1.05, 1.06)	<0.001
2009m1	1.02 (1.01, 1.02)	1.06 (1.05, 1.06)	<0.001
2009m2	1.02 (1.01, 1.02)	1.06 (1.05, 1.06)	<0.001
2009m3	1.02 (1.01, 1.02)	1.06 (1.05, 1.06)	<0.001
2009m4	1.02 (1.01, 1.02)	1.06 (1.05, 1.06)	<0.001
2009m5	1.02 (1.01, 1.02)	1.06 (1.05, 1.06)	<0.001
2009m6	1.02 (1.01, 1.02)	1.06 (1.05, 1.06)	<0.001
2009m7	1.02 (1.01, 1.02)	1.06 (1.05, 1.06)	<0.001
2009m8	1.02 (1.01, 1.02)	1.06 (1.05, 1.06)	<0.001
2009m9	1.02 (1.01, 1.02)	1.06 (1.05, 1.07)	<0.001
2009m10	1.02 (1.01, 1.02)	1.06 (1.05, 1.07)	<0.001
2009m11	1.02 (1.01, 1.02)	1.06 (1.05, 1.07)	<0.001
2009m12	1.02 (1.02, 1.02)	1.06 (1.06, 1.07)	<0.001
2010m1	1.02 (1.02, 1.02)	1.06 (1.06, 1.07)	<0.001
2010m2	1.02 (1.02, 1.02)	1.06 (1.06, 1.07)	<0.001
2010m3	1.02 (1.02, 1.02)	1.06 (1.06, 1.07)	<0.001
2010m4	1.02 (1.02, 1.02)	1.06 (1.06, 1.07)	<0.001
2010m5	1.02 (1.02, 1.02)	1.06 (1.06, 1.07)	<0.001
2010m6	1.02 (1.02, 1.02)	1.06 (1.06, 1.07)	<0.001
2010m7	1.02 (1.02, 1.02)	1.07 (1.06, 1.07)	<0.001
2010m8	1.02 (1.02, 1.02)	1.07 (1.06, 1.07)	<0.001
2010m9	1.02 (1.02, 1.02)	1.07 (1.06, 1.07)	<0.001
2010m10	1.02 (1.02, 1.02)	1.07 (1.06, 1.07)	<0.001
2010m11	1.02 (1.02, 1.02)	1.07 (1.06, 1.08)	<0.001
2010m12	1.02 (1.02, 1.02)	1.07 (1.06, 1.08)	<0.001
2011m1	1.02 (1.02, 1.02)	1.07 (1.06, 1.08)	<0.001
2011m2	1.02 (1.02, 1.02)	1.07 (1.06, 1.08)	<0.001
2011m3	1.02 (1.02, 1.02)	1.07 (1.06, 1.08)	<0.001
2011m4	1.02 (1.02, 1.02)	1.07 (1.06, 1.08)	<0.001
2011m5	1.02 (1.02, 1.02)	1.07 (1.06, 1.08)	<0.001
2011m6	1.02 (1.02, 1.02)	1.07 (1.07, 1.08)	<0.001
2011m7	1.02 (1.02, 1.02)	1.07 (1.07, 1.08)	<0.001
2011m8	1.02 (1.02, 1.02)	1.07 (1.07, 1.08)	<0.001
2011m9	1.02 (1.02, 1.02)	1.08 (1.07, 1.08)	<0.001
2011m10	1.02 (1.02, 1.02)	1.08 (1.07, 1.08)	<0.001
2011m11	1.02 (1.02, 1.02)	1.08 (1.07, 1.09)	<0.001
2011m12	1.02 (1.02, 1.02)	1.08 (1.07, 1.09)	<0.001
2012m1	1.02 (1.02, 1.02)	1.08 (1.07, 1.09)	<0.001
2012m2	1.02 (1.02, 1.02)	1.08 (1.07, 1.09)	<0.001
2012m3	1.02 (1.02, 1.02)	1.08 (1.07, 1.09)	<0.001
2012m4	1.02 (1.02, 1.02)	1.08 (1.07, 1.09)	<0.001
2012m5	1.02 (1.02, 1.02)	1.08 (1.07, 1.09)	<0.001
2012m6	1.02 (1.02, 1.03)	1.08 (1.07, 1.09)	<0.001
2012m7	1.02 (1.02, 1.03)	1.09 (1.07, 1.10)	<0.001

Month of change	Trend in first period (annual IRR (95%CI))	Trend in second period (annual IRR (95%CI))	p-value for difference in trends
2012m8	1.02 (1.02, 1.03)	1.09 (1.08, 1.10)	<0.001
2012m9	1.02 (1.02, 1.03)	1.09 (1.08, 1.10)	<0.001
2012m10	1.02 (1.02, 1.03)	1.09 (1.08, 1.10)	<0.001
2012m11	1.02 (1.02, 1.03)	1.09 (1.08, 1.10)	<0.001
2012m12	1.02 (1.02, 1.03)	1.09 (1.08, 1.10)	<0.001
2013m1	1.02 (1.02, 1.03)	1.09 (1.08, 1.10)	<0.001
2013m2	1.02 (1.02, 1.03)	1.09 (1.08, 1.11)	<0.001
2013m3	1.02 (1.02, 1.03)	1.10 (1.08, 1.11)	<0.001
2013m4	1.02 (1.02, 1.03)	1.10 (1.08, 1.11)	<0.001
2013m5	1.02 (1.02, 1.03)	1.10 (1.08, 1.11)	<0.001
2013m6	1.02 (1.02, 1.03)	1.10 (1.09, 1.11)	<0.001
2013m7	1.02 (1.02, 1.03)	1.10 (1.09, 1.12)	<0.001
2013m8	1.02 (1.02, 1.03)	1.10 (1.09, 1.12)	<0.001
2013m9	1.02 (1.02, 1.03)	1.10 (1.09, 1.12)	<0.001
2013m10	1.03 (1.02, 1.03)	1.11 (1.09, 1.12)	<0.001
2013m11	1.03 (1.02, 1.03)	1.11 (1.09, 1.12)	<0.001
2013m12	1.03 (1.02, 1.03)	1.11 (1.09, 1.13)	<0.001
2014m1	1.03 (1.02, 1.03)	1.11 (1.09, 1.13)	<0.001
2014m2	1.03 (1.02, 1.03)	1.11 (1.09, 1.13)	<0.001
2014m3	1.03 (1.02, 1.03)	1.12 (1.10, 1.14)	<0.001
2014m4	1.03 (1.02, 1.03)	1.12 (1.10, 1.14)	<0.001
2014m5	1.03 (1.02, 1.03)	1.12 (1.10, 1.14)	<0.001
2014m6	1.03 (1.02, 1.03)	1.12 (1.10, 1.15)	<0.001
2014m7	1.03 (1.02, 1.03)	1.13 (1.10, 1.15)	<0.001
2014m8	1.03 (1.02, 1.03)	1.13 (1.10, 1.15)	<0.001
2014m9	1.03 (1.02, 1.03)	1.13 (1.11, 1.16)	<0.001
2014m10	1.03 (1.02, 1.03)	1.14 (1.11, 1.16)	<0.001
2014m11	1.03 (1.02, 1.03)	1.14 (1.11, 1.17)	<0.001
2014m12	1.03 (1.03, 1.03)	1.14 (1.11, 1.17)	<0.001
2015m1	1.03 (1.03, 1.03)	1.15 (1.11, 1.18)	<0.001
2015m2	1.03 (1.03, 1.03)	1.15 (1.12, 1.19)	<0.001
2015m3	1.03 (1.03, 1.03)	1.15 (1.12, 1.19)	<0.001
2015m4	1.03 (1.03, 1.03)	1.16 (1.12, 1.20)	<0.001
2015m5	1.03 (1.03, 1.03)	1.16 (1.12, 1.20)	<0.001
2015m6	1.03 (1.03, 1.03)	1.17 (1.12, 1.21)	<0.001
2015m7	1.03 (1.03, 1.03)	1.17 (1.12, 1.22)	<0.001
2015m8	1.03 (1.03, 1.03)	1.18 (1.13, 1.23)	<0.001
2015m9	1.03 (1.03, 1.03)	1.19 (1.13, 1.25)	<0.001
2015m10	1.03 (1.03, 1.03)	1.20 (1.14, 1.26)	<0.001
2015m11	1.03 (1.03, 1.03)	1.21 (1.14, 1.28)	<0.001
2015m12	1.03 (1.03, 1.03)	1.23 (1.15, 1.31)	<0.001
2016m1	1.03 (1.03, 1.03)	1.25 (1.16, 1.33)	<0.001
2016m2	1.03 (1.03, 1.03)	1.27 (1.17, 1.37)	<0.001
2016m3	1.03 (1.03, 1.03)	1.29 (1.19, 1.41)	<0.001
2016m4	1.03 (1.03, 1.03)	1.32 (1.20, 1.46)	<0.001
2016m5	1.03 (1.03, 1.03)	1.36 (1.21, 1.52)	<0.001
2016m6	1.03 (1.03, 1.03)	1.40 (1.23, 1.59)	<0.001
2016m7	1.03 (1.03, 1.03)	1.45 (1.25, 1.69)	<0.001

Month of change	Trend in first period (annual IRR (95%CI))	Trend in second period (annual IRR (95%CI))	p-value for difference in trends
2016m8	1.03 (1.03, 1.03)	1.51 (1.26, 1.82)	<0.001
2016m9	1.03 (1.03, 1.03)	1.58 (1.26, 1.99)	<0.001
2016m10	1.03 (1.03, 1.03)	1.71 (1.28, 2.29)	<0.001
2016m11	1.03 (1.03, 1.03)	1.90 (1.28, 2.82)	0.002
2016m12	1.03 (1.03, 1.03)	2.28 (1.28, 4.05)	0.007
2017m1	1.03 (1.03, 1.03)	3.24 (1.24, 8.44)	0.019
2017m2	1.03 (1.03, 1.03)	8.06 (0.96, 67.92)	0.059
2017m3	1.03 (1.03, 1.03)	-	-
