# Supplementary material for: Investigation of the impact of the NICE guidelines regarding antibiotic prophylaxis during invasive dental procedures on the incidence of infective endocarditis in England: an electronic health records study
Source: BMC Med. 2020 Apr 2;18:84. doi: 10.1186/s12916-020-01531-y (PMC7114779; doi:10.1186/s12916-020-01531-y)

Investigation of the impact of the NICE guidelines regarding antibiotic prophylaxis during invasive dental procedures on the incidence of infective endocarditis in England: an Electronic Health Records study - Supplementary figures

Figure S1. Effect of applying different methods to adjust for changes in population

(A) Criteria A

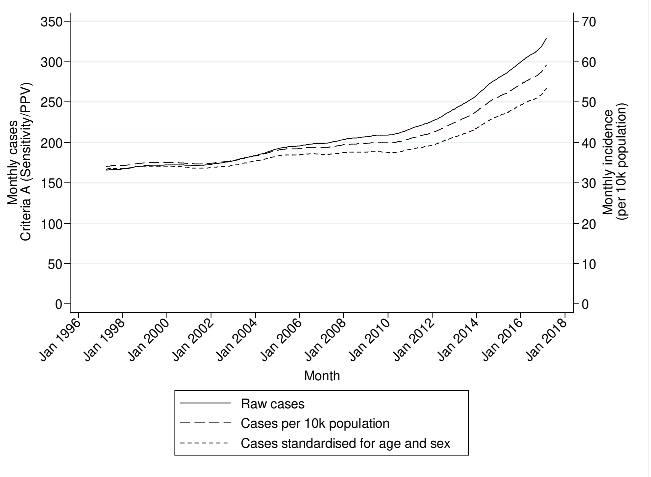

(B) Criteria B

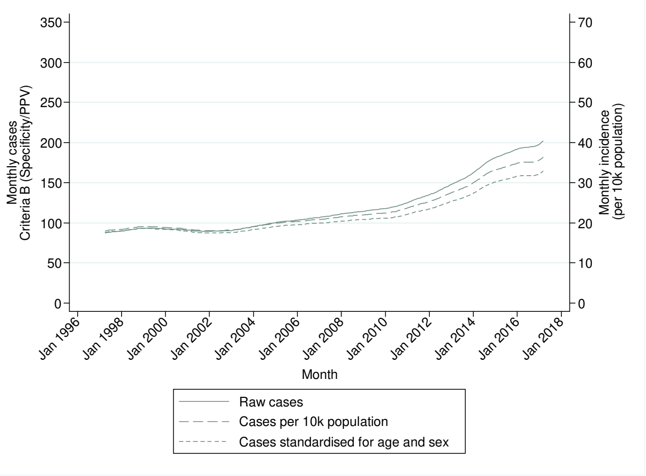

(C) Criteria C

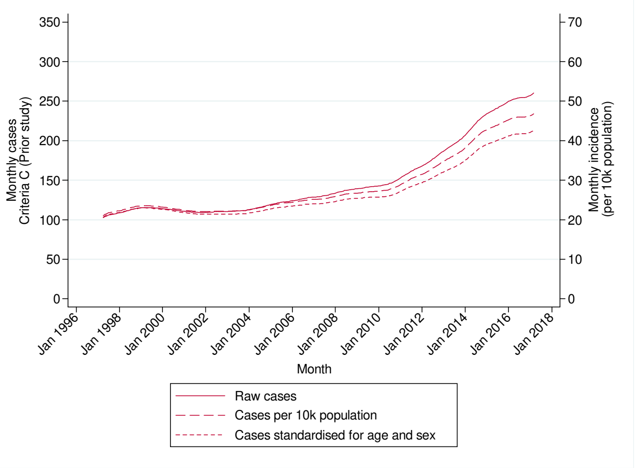

Figure S2. Monthly cases of infective endocarditis *excluding* individuals identified as high-risk or as illicit drug users

(A) excluding individuals identified as high-risk

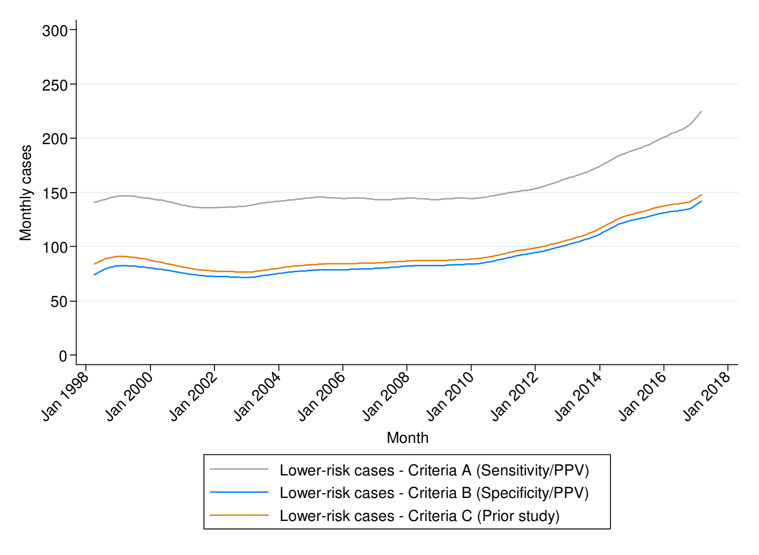

(B) excluding individuals with a recorded history of illicit drug use

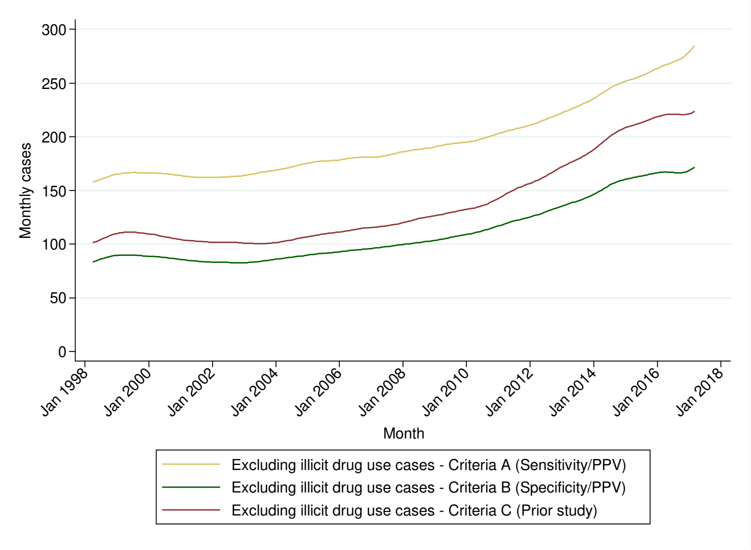

Figure S3. Causative organism based on secondary diagnosis codes in HES

(A) Criteria A

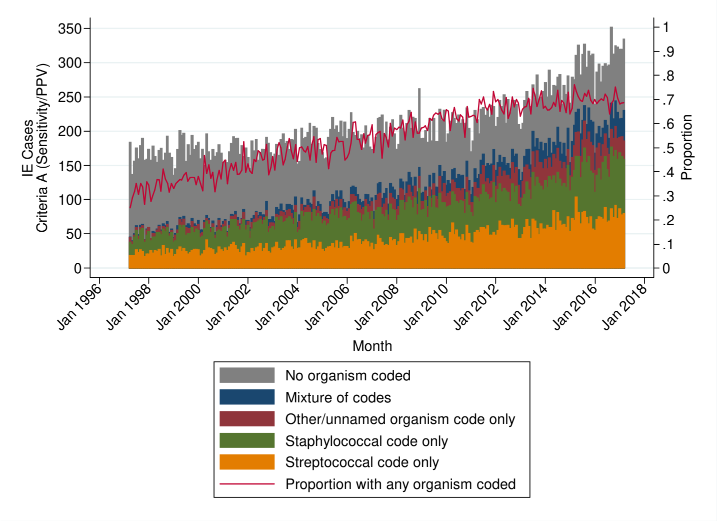

(B) Criteria B

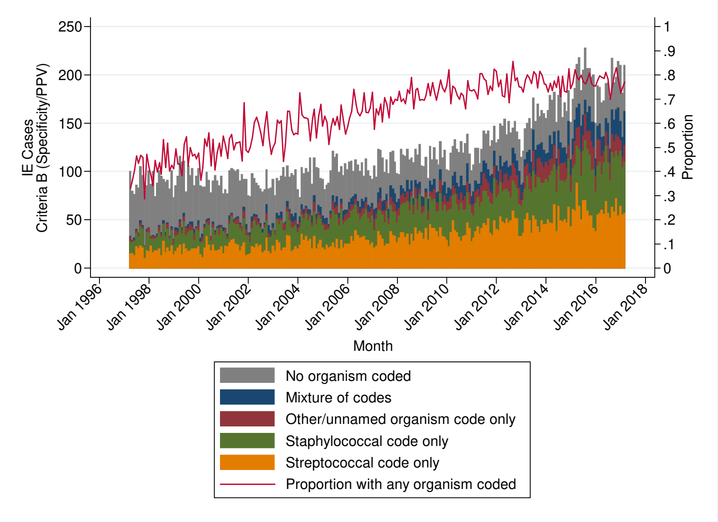

(C) Criteria C

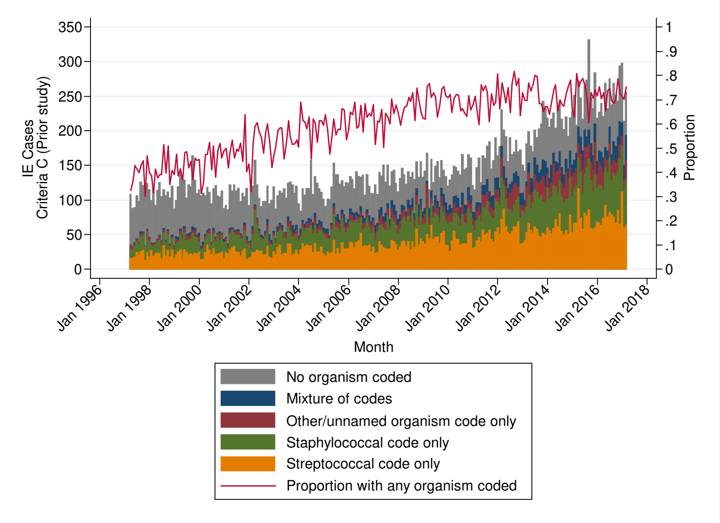

Figure S4. Of infective endocarditis cases with an organism code present in HES, proportion that were coded as Streptococcal, Staphylococcal, or Other/Unnamed (including mixtures)

(A) Criteria A

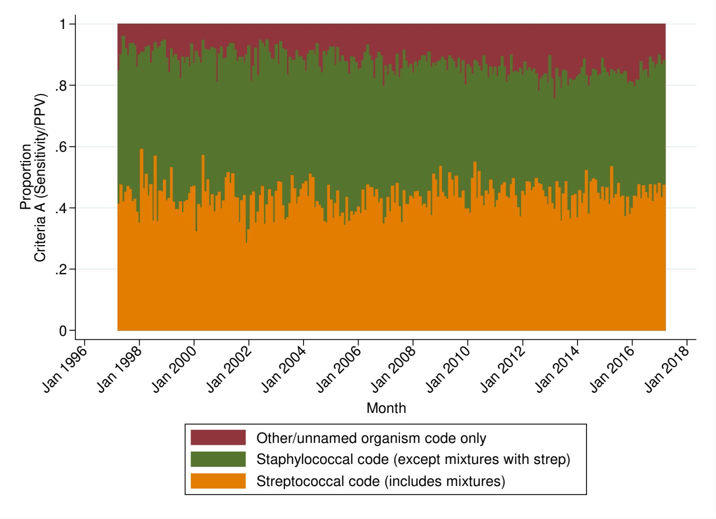

(B) Criteria B

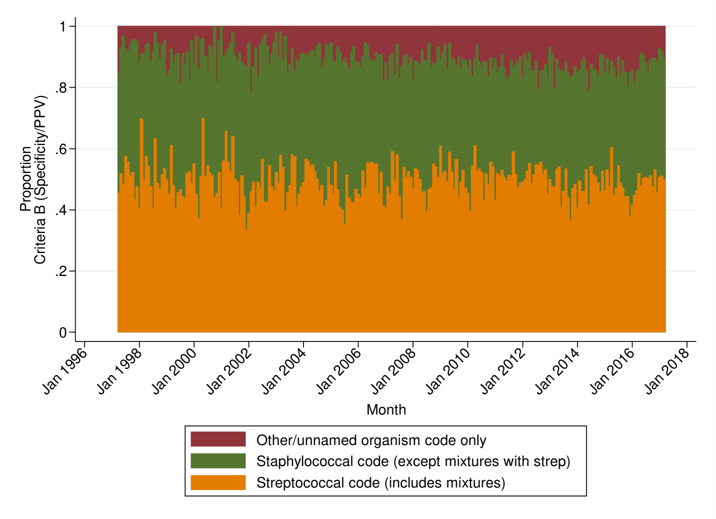

(C) Criteria C

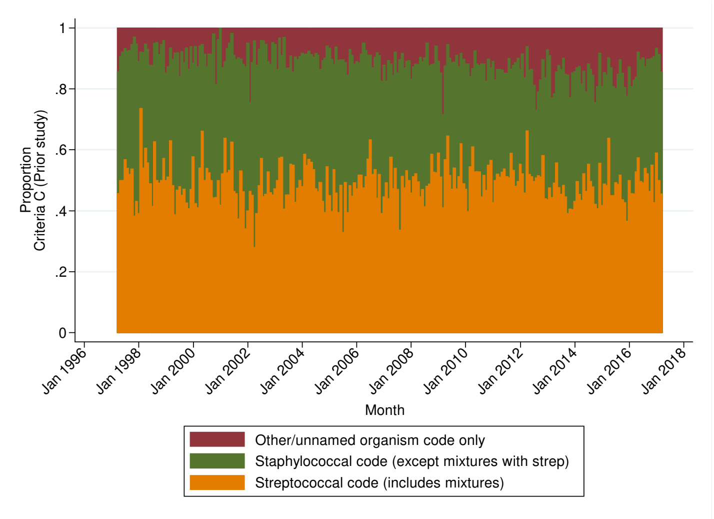

Figure S5. Of infective endocarditis cases with an organism code present in HES, proportion that were coded exclusively as Streptococcal, Staphylococcal or Other/Unnamed, or else with a mixture of codes

(A) Criteria A

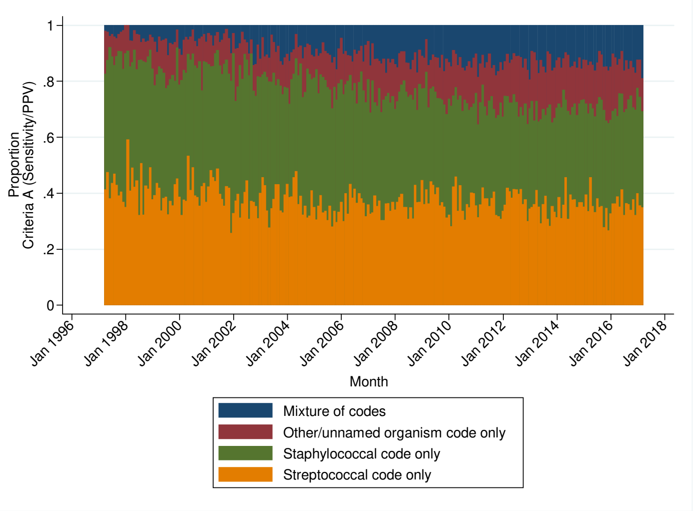

(B) Criteria B

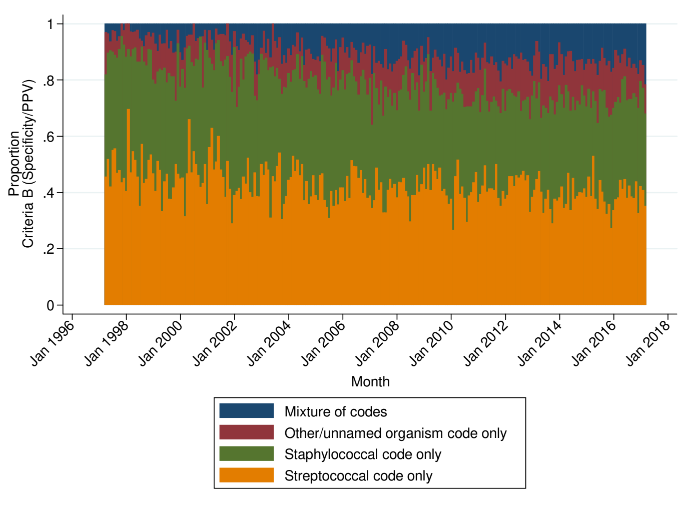

(C) Criteria C

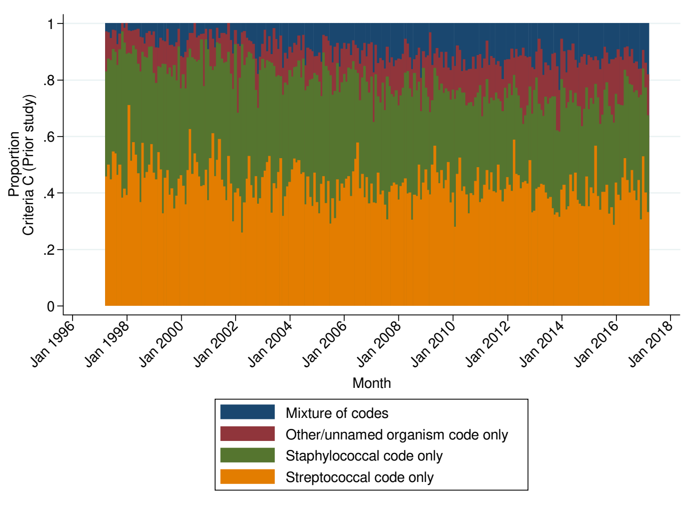

Figure S6. Causative organism based on SGSS: monthly agreement of SGSS organism compared to HES organism code, based on 3 groups: Streptococcal, Staphylococcal, Other/unnamed

(A) Criteria A

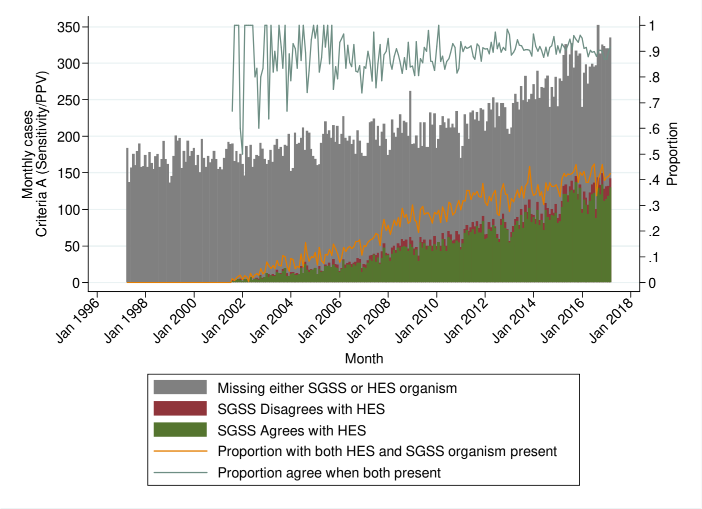

(B) Criteria B

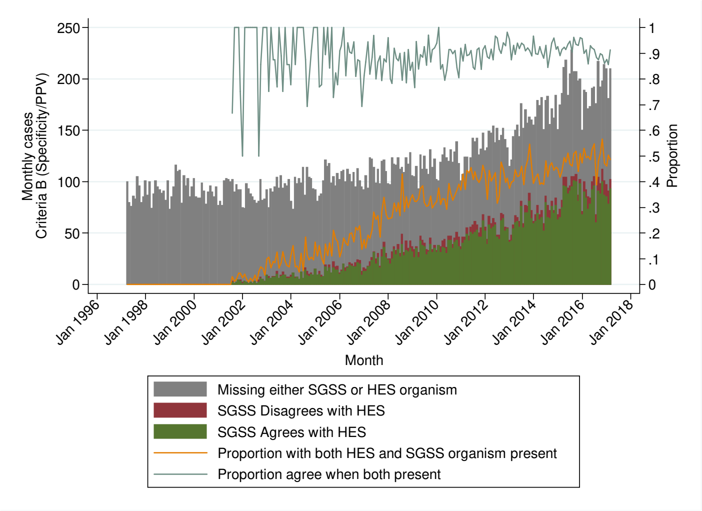

(C) Criteria C

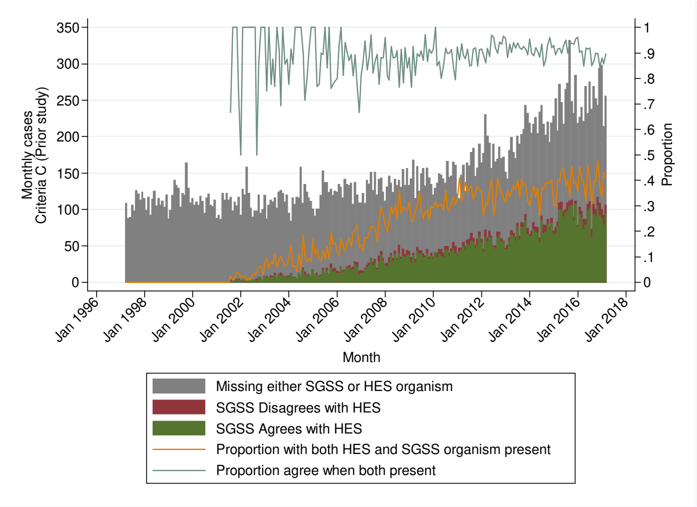

Figure S7. Of all infective endocarditis cases that were matched to an organism in SGSS, proportion that were classed as oral streptococci

(A) Criteria A

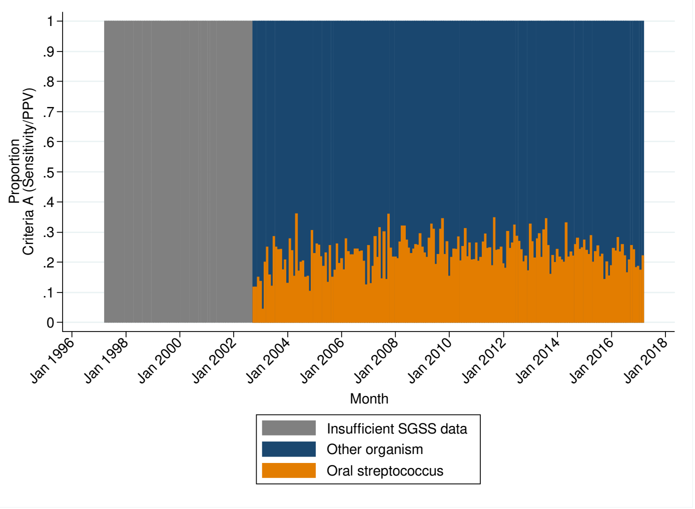

(B) Criteria B

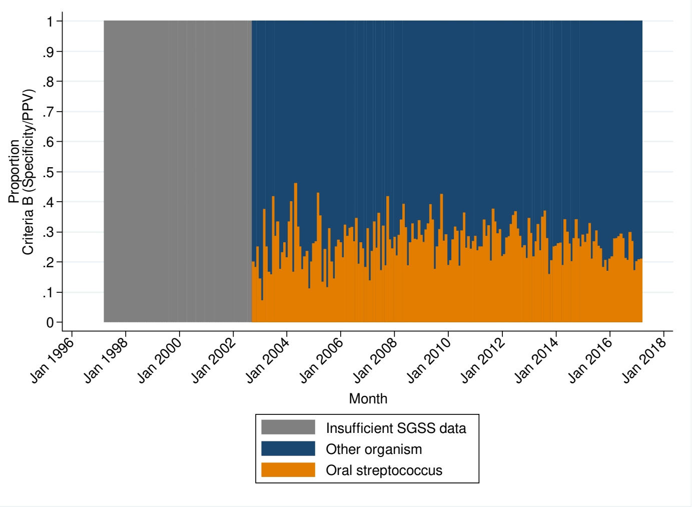

(C) Criteria C

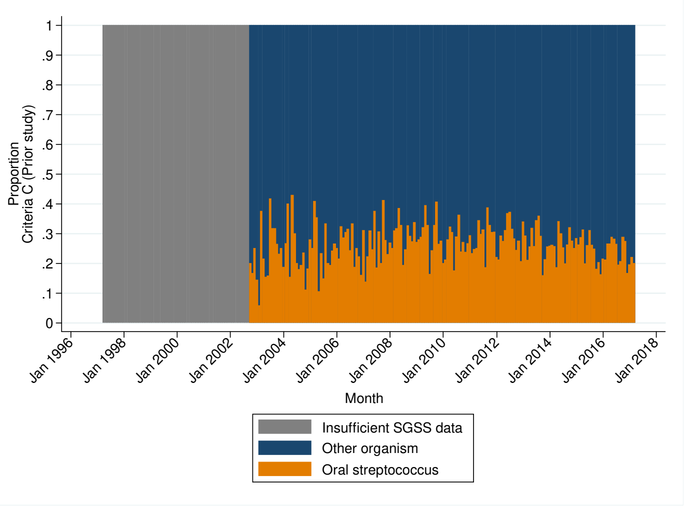

Figure S8. Infective endocarditis cases that were matched to an organism in SGSS. The HACEK group consists of *Haemophilus* species, *Aggregatibacter* (previously *Actinobacillus*), *Cardiobacterium*, *Eikenella*, *Kingella*. CONS – Coagulase negative staphylococci.

(A) Criteria A

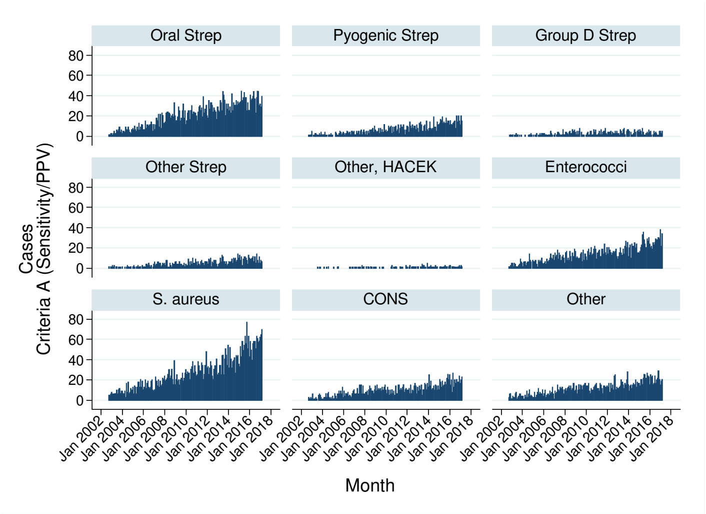

(B) Criteria B

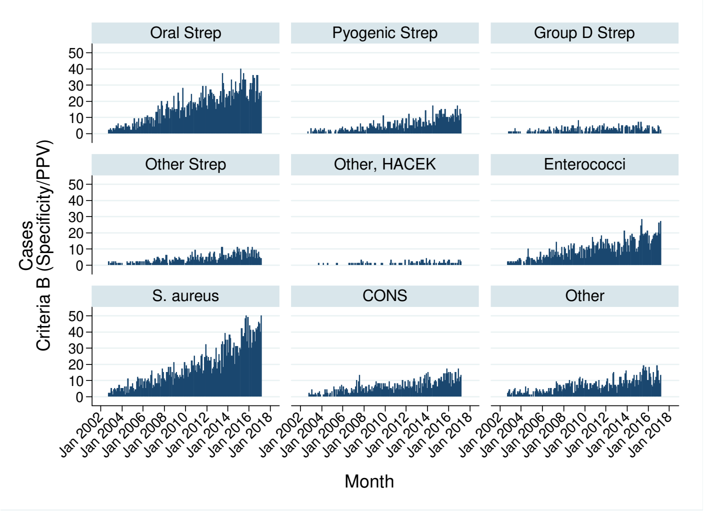

(C) Criteria C

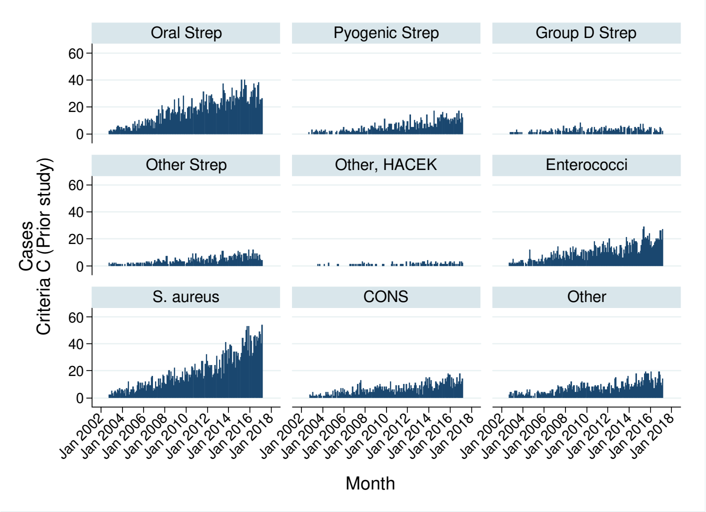

Supplement: Supplementary file 2 — Supplementary figures. All supplementary figures to accompany the manuscript. Figure S1. Effect of applying different methods to adjust for changes in population, for all 3 criteria. Figure S2. Monthly cases of infective endocarditis excluding individuals identified as high-risk or as illicit drug users. Figure S3. Causative organism based on secondary diagnosis codes in HES, for all 3 criteria. Figure S4. Of infective endocarditis cases with an organism code present in HES, proportion that were coded as streptococcal, staphylococcal, or other/unnamed (including mixtures), for all 3 criteria. Figure S5. Of infective endocarditis cases with an organism code present in HES, proportion that were coded exclusively as streptococcal, staphylococcal or other/unnamed, or else with a mixture of codes, for all 3 criteria. Figure S6. Causative organism based on SGSS: monthly agreement of SGSS organism compared to HES organism code, based on 3 groups: streptococcal, staphylococcal, other/unnamed, for all 3 criteria. Figure S7. Of all infective endocarditis cases that were matched to an organism in SGSS, proportion that were classed as oral streptococci, for all 3 criteria. Figure S8. Infective endocarditis cases that were matched to an organism in SGSS. The HACEK group consists of Haemophilus species, Aggregatibacter (previously Actinobacillus), Cardiobacterium, Eikenella, Kingella. CONS – Coagulase negative staphylococci. (PDF 231 kb) [file 12916_2020_1531_MOESM2_ESM.pdf]
